# The impact of inflammation, neuromodulation, and gut microbiota on developing cardiac fibrosis and hypertension

**DOI:** 10.1093/cvr/cvag054

**Published:** 2026-02-27

**Authors:** Michał Kozdrowicki, Piotr Szczepaniak, Vladyslav Kyslyi, Lorenzo Carnevale, Daniela Carnevale, Giuseppe Lembo, Tomasz J Guzik, Tomasz P Mikołajczyk

**Affiliations:** Department of Internal and Agricultural Medicine, Jagiellonian University Medical College, Wrocławska 1-3, Krakow 30-901, Poland; Department of Internal and Agricultural Medicine, Jagiellonian University Medical College, Wrocławska 1-3, Krakow 30-901, Poland; Department of Internal and Agricultural Medicine, Jagiellonian University Medical College, Wrocławska 1-3, Krakow 30-901, Poland; Department of Angiocardioneurology and Translational Medicine, IRCCS Neuromed, Pozzilli (IS), Italy; Department of Molecular Medicine, ‘Sapienza’ University of Rome, Rome, Italy; Department of Angiocardioneurology and Translational Medicine, IRCCS Neuromed, Pozzilli (IS), Italy; Department of Medical and Surgical Sciences and Biotechnologies, Polo Pontino, ‘Sapienza’ University of Rome, Rome, Italy; Department of Angiocardioneurology and Translational Medicine, IRCCS Neuromed, Pozzilli (IS), Italy; Department of Molecular Medicine, ‘Sapienza’ University of Rome, Rome, Italy; Department of Internal and Agricultural Medicine, Jagiellonian University Medical College, Wrocławska 1-3, Krakow 30-901, Poland; BHF Centre for Research Excellence, Centre for Cardiovascular Sciences, The University of Edinburgh, Edinburgh, UK; Department of Internal and Agricultural Medicine, Jagiellonian University Medical College, Wrocławska 1-3, Krakow 30-901, Poland

**Keywords:** Heart failure, Cardiac fibrosis, Inflammation, Neuromodulation, Diet

## Abstract

Cardiovascular diseases (CVD) are the leading cause of premature mortality worldwide. Due to pressure overload and cardiac fibrosis, CVD often begin with hypertension and gradually progress to heart failure. Cardiac fibrosis reduces the number of functional cardiomyocytes and the force of contraction while increasing oxygen demand. It has been noted that myofibroblasts, which produce excessive amounts of extracellular matrix in the failing heart, express specific proteins such as periostin, tenascin C, thrombospondin, and osteopontin. Their activation involves immune cells that have a well-documented effect on the pathogenesis of hypertension.

Moreover, dysregulation of the autonomic nervous system and sympathetic hyperactivity heightens peripheral inflammation and fosters fibrosis. In this review, we outline and summarize the most significant and recent findings concerning the molecular pathways of immune activation, neuromodulation, epigenetic modifications, and the impact of gut microbiota on myofibroblast activation and fibrosis in the heart, as well as potential therapeutic options (e.g. experimental anti-inflammatory treatments, epigenetic modulators, and vagus nerve stimulation). We will also highlight how current heart failure treatments, including renin-angiotensin-aldosterone system (RAA) inhibitors, β-adrenergic receptor (β-AR) antagonists, sodium-glucose co-transporter 2 (SGLT2) inhibitors, the Dietary Approaches to Stop Hypertension (DASH), and the Mediterranean diet, affect these processes at a molecular level. A comprehensive understanding of the neuroimmune mechanisms involved in the pathogenesis of heart failure and hypertension is particularly crucial in light of the increased risk of CVD following the COVID-19 pandemic, which resulted from the ‘cytokine storm’ during SARS-CoV-2 infection.

## 1. Introduction

Cardiovascular diseases (CVD), which primarily include coronary heart disease, stroke, thromboembolism, atherosclerosis, hypertension, arrhythmias, and heart failure (HF) are the leading cause of death worldwide and constitute a significant financial burden for public health. They commonly cause reduced efficiency and quality of life, requiring long-term care and chronic treatment.^1^ CVD were estimated to cost the European Union €282 billion annually, which translates to €630 per person.^2^ CVD most often occur in older people, but recently, they have become more common in younger people due to obesity, unhealthy lifestyles, including chronic stress and limited physical activity. Annual global premature CVD deaths (age cut-offs ranging from 50 to 65 years) increased by 25% between 1990 and 2019.^3^ Moreover, the COVID-19 pandemic has caused an increase in cardiovascular complications.^4–6^ For this reason, intensive research is being conducted to improve the early detection of diseases and more effective treatment.

One of the essential factors that determines the development of other CVD is poorly controlled hypertension. It has been estimated that 30% of adults worldwide have hypertension, of which only about 20% have it well controlled.^7–9^ As with other CVD, the incidence is much higher in older people (>70% of adults have hypertension over the age of 70). Between 1990 and 2019, the number of people with hypertension doubled and is approximately 1.28 billion patients globally.^9^ Poorly controlled hypertension causes endothelial damage, impaired vessel relaxation, increased vascular stiffness and leads to the development of atherosclerosis.^10,11^ Increased afterload causes cardiomyocyte hypertrophy and fibroblast activation, resulting in left ventricular remodelling and dysfunction, impaired perfusion, and electrical disturbances.^12^ In addition, hypertension leads to several organ complications, including damage and fibrosis of the renal glomeruli, retinal ischaemia, and haemorrhages. It disrupts cerebral circulation, increasing the risk of strokes fourfold.^13,14^ The control of hypertension has improved significantly after the introduction of several groups of antihypertensive drugs, such as diuretics, renin-angiotensin-aldosterone (RAA) inhibitors, beta-adrenergic receptor (β-AR) antagonists, and calcium channel blockers. Nevertheless, normalization of the blood pressure is not achieved in 10–30% of treated hypertensive patients. In this group of patients, in addition to adding other groups of drugs (e.g. aldosterone antagonists), non-pharmacological methods such as renal denervation and carotid baroreceptor stimulation are tested in studies.^15^ In clinical trials, baxdrostat, an aldosterone synthase inhibitor, shows promising results.^16^

Understanding the molecular and cellular mechanisms leading to the development of hypertension, is essential, especially because many of them also contribute to its complications, such as myocardial remodelling and fibrosis, which overall worsen the prognosis in patients with CVD. The activation of fibroblasts, increased production of extracellular matrix (ECM) and type I collagen and reduced activity of metalloproteinases impair the function of cardiomyocytes, leading to HF. The RAA system and AR receptors are involved in these processes, and their targeting by antihypertensive drugs directly inhibit fibrosis.^17^ Secondly, immune system activation plays a significant role in myocardial remodelling. It has been shown that myocardial fibrosis is accompanied by the infiltration of activated T lymphocytes, polarization of macrophages, higher secretion of pro-inflammatory cytokines such as IL-1β, IL-6, TNF-α, TGF-β, or chemokines such as CCL2, CXCL12, which play an essential role in the pathogenesis of hypertension.^18,19^ Thirdly, in the fibrosis processes, we observe dysregulation of the autonomic nervous system, with the dominant activity of the sympathetic part identical to that in hypertension.^20^ Fourthly, changes in the expression of similar genes were observed in people with hypertension and myocardial fibrosis.^21^ Fifthly, the influence of gut microbiota and its metabolites on fibroblast activation and the brain-heart-gut axis is worth noting. Diet is an essential element in the treatment of hypertension. A high-fibre diet increases the diversity of the gut microbiome, the amount of short-chain fatty acids (SCFA) and hydrogen sulfide (H_2_S) and reduces hypertension and fibrosis.^22^ In contrast, a high-choline diet, through the increase in trimethylamine N-oxide (TMAO) levels, exacerbates hypertension and HF.^23^

The role of the immune system, neuromodulation, epigenetic regulation of gene expression, and gut microflora changes are undoubtedly important research areas in the pathogenesis of CVD, particularly myocardial fibrosis and hypertension. This review will summarize and organize the findings, highlighting the latest discoveries.

## 2. Inflammation in hypertension

Theories regarding the role of immune system activation in the pathogenesis of hypertension have developed significantly over the last 20 years. Attempts to better understand the mechanisms were initiated by the discovery by Guzik *et al*., where mice lacking recombination activating gene 1 (RAG1), which causes a lack of T and B lymphocytes, do not develop hypertension despite infusion of Ang II, aldosterone or a high-salt diet.^18^ However, the injection of T lymphocytes from wild-type male mice to RAG1^−/−^ mice restored the mechanisms of hypertension.^24^

Many observational studies have noted an increased risk of hypertension in inflammatory and autoimmune diseases (IADs). A recent meta-analysis of 122 studies showed that patients with IADs have a 36% increased risk of developing hypertension and other CVD, such as ischaemic heart disease, HF, cerebrovascular disease, and 2.5 times greater risk of atherosclerotic plaque presence.^25^ In this meta-analysis, in systemic lupus erythematosus (SLE), the risk of hypertension was increased by 3.4-fold, and in Sjogren's syndrome (SS) by 2-fold.^25^ In patients with periodontitis, the risk of hypertension increases by 22%,^26^ with rheumatoid arthritis (RA) by 45%,^27^ while psoriatic arthritis increases this risk by 90%.^28^ Compared to the general population, patients with inflammatory bowel disease (IBD) also had an increased risk of hypertension—7.7% in Crohn's disease and 10.9% in ulcerative colitis.^29^ Hypertension is also a complication of infectious diseases. SARS-CoV-2 virus infection may contribute to uncontrolled excessive activation of the immune system and cytokine storm, which is one of the leading causes of poor prognosis and mortality due to COVID-19.^30^ In a meta-analysis by Li *et al*., hypertension increases the risk of mortality from COVID-19 by 19%.^31^ Patients who had COVID-19 had a higher risk of developing hypertension, which is one of the components of the post-COVID syndrome.^32^ There are mixed results from studies of people living with HIV (PLHIV). Some studies indicate an increased risk of hypertension compared to healthy individuals as a consequence of chronic inflammation and antiretroviral therapy. Others suggest a reduced risk due to humoral immunity and lymphocyte function disorders. Results from a meta-analysis of studies involving over 11 million participants showed variation across continents. PLHIV had a slightly increased risk of hypertension in North America but a lower risk in Africa and Asia.^33^

On the other hand, multiple studies have shown that hypertension triggers immune system activation in individuals without IADs. Hypertensive patients without IADs have elevated levels of CRP and pro-inflammatory cytokines such as IL-1β, IL-6, and TNF-α,^34^ as well as increased numbers of circulating lymphocytes, monocytes, and neutrophils in peripheral blood.^35^ An elevated neutrophil-to-lymphocyte ratio increases the risk of hypertension.^36^ In animal models of hypertension, levels of inflammatory chemokines are also elevated, including CCL2, CCL5 (RANTES), CXCL9, and CXCL10.^37^

### 2.1 Innate immunity

Studies conducted so far provide key changes in the activation of both innate immunity (by monocytes, macrophages, and dendritic cells (DCs)) and adaptive immune responses (by specific subpopulations of T and B lymphocytes). Foreign antigens, including pathogen-associated molecular patterns (PAMPS) and damage-associated molecular patterns (DAMPS), which interact with Toll-like receptors (TLR), activate the NLRP3 (nucleotide oligomerisation domain-like receptor family pyrin domain containing 3) inflammasome. Activation of the NLRP3 inflammasome results in increased production of IL-1β, IL-18, caspase-1, and activation of NADPH oxidase (nicotinamide adenine dinucleotide phosphate), which leads to increased production of reactive oxygen species (ROS). This contributes to lipid peroxidation and accumulation of isolevuglandins (isoLGs), which, by binding to antigen-presenting cells (APC), act as neoantigens and activate the immune response observed in hypertension.^38^ Rats with hypertension had increased expression of TLR2 and TLR4 in the heart, vessels, and brain^39^ while blocking the NLRP3 inflammasome in mice reduced the production of IL-1β and IL-18 and protected against the development of DOCA–salt-induced hypertension.^40^ It has been shown that key compounds in the pathogenesis of hypertension, such as endothelin, angiotensin II, and aldosterone, activate the NLRP3 inflammasome,^41^ and NLRP3 gene polymorphisms are associated with hypertension.^42^

Another essential element of innate immunity is the differentiation of monocytes into macrophages or DCs, which, as APCs, can present antigens and activate lymphocytes. The hypertensive stretch of human aortic epithelial cells caused an increase in the secretion of pro-inflammatory cytokines, resulting in the transformation of resting monocytes (CD14++/CD16−) into a pro-inflammatory phenotype (CD14+/CD16++) and an increase in the expression of CD209, a marker of activated macrophages and DCs.^43^ Another critical factor is the high concentration of sodium, which flows into DCs through the epithelial sodium channel (ENaC) and is exchanged for calcium, which activates NADPH oxidase, increasing ROS and isoLGs. It has been shown that mice on a high-salt diet have a more significant macrophage infiltrate in the kidneys, with a dominant pro-inflammatory M1 phenotype.^44^ Moreover, CD11c+ monocyte-derived dendritic cells from mice on a high-salt diet show increased isoLG adducts. Inhibiting serum-glucocorticoid-regulated kinase-1 (SGK1) in dendritic cells, which regulate ENaC levels, protects against salt-sensitive hypertension.^45^ Salt-sensitive hypertension and monocyte activation are also associated with increased sympathetic activation in the forebrain lamina terminalis via prostaglandin E2 receptor (EP3) activation. EP3 deficiency reduced isoLG accumulation, DC activation, and sympathetic stimulation.^46^

### 2.2 Adaptive immunity

**Figure 1:**
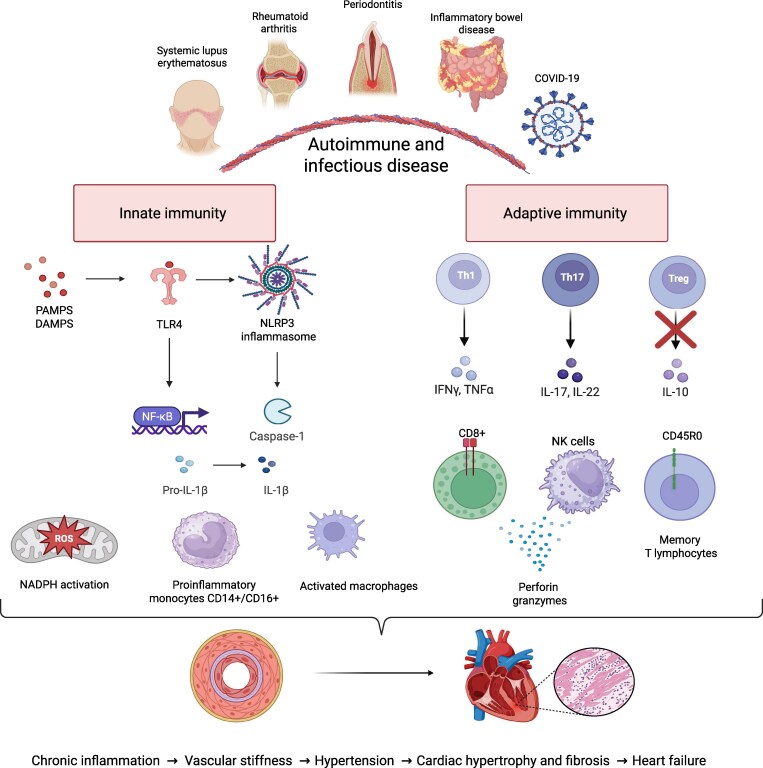
Activation of innate and adaptive immunity in IADs diseases leads to hypertension development and CVD. Chronic inflammation, activation of TLR receptors, NLRP3 inflammasome, and NF-κB increase expression of pro-inflammatory cytokines, oxidative stress and ROS production, macrophage activation, and secondary adaptive immunity mechanisms. During hypertension, an increase in Th1, Th17, and Tc CD8+ cytotoxic lymphocytes content and a reduced level of anti-inflammatory Treg lymphocytes were observed. As a consequence, it leads to further aggravation of hypertension and pressure overload of the heart and stimulates fibrosis, which increases heart failure. This figure has been created with Biorender.com. IADs, inflammatory and autoimmune diseases; PAMPS, pathogen-associated molecular patterns; DAMPS, damage-associated molecular patterns; NLRP3, nucleotide oligomerisation domain-like receptor family pyrin domain containing 3; TLR, Toll-like receptors; NF-κB, nuclear factor kappa-light-chain-enhancer of activated B cells; NK, natural killer cells; NADPH, nicotinamide adenine dinucleotide phosphate; ROS, reactive oxygen species; IFN-γ, interferon γ; TNF-α, tumour necrosis factor α.

In studies on animal models and peripheral blood from humans with hypertension, significant disproportions in subpopulations of lymphocytes have been observed. In patients with hypertension, an increase in the percentage of γδ T lymphocytes, cytotoxic Tc lymphocytes (CD8 + CD57 + CD28null), memory T lymphocytes (CD45 + RO), and natural killer cells (NKc) was observed in the blood, which correlated with increased secretion of granzymes, perforins, IFN-y and TNF-α.^47,48^ In mice, depletion of γδ T or Tc (CD8^−/−^) lymphocytes protected against the development of hypertension.^49^ In experimental models of hypertension in mice, the increased perivascular infiltration of Th17 lymphocytes, CD4+ T cells, and CD3 + CD4−CD8− T lymphocytes was observed. This was accompanied by elevated IL-17 production.^50^ Interestingly, the transfer of Th17 lymphocytes caused exacerbation of hypertension.^51^ In turn, the transferring T regulatory lymphocytes (CD4 + CD25 + FoxP3) secreting immunosuppressive IL-10 reduced cardiovascular complications.^52,53^ Additionally, the imbalance between Th17/Treg infiltration and increased IL-17, TNF-α, and decreased IL-10 in mice was observed in the heart, kidney, and spleen of Ang II-induced hypertensive mice.^54^ The areas of immune system activation leading to the development of hypertension are shown in *Figure 1*.

### 2.3 Autonomic nervous system

In hypertension, the immune system cooperates with the autonomic nervous system (ANS). Ang II injection stimulates sympathetic innervation of the spleen, promoting the release of activated T lymphocytes. Denervation of the coeliac ending of the vagus nerve or blockade of the α7 nicotinic acetylcholine receptor (α7nAChR) protect against the development of hypertension after Ang II infusion.^55,56^ Similar effects were caused by damage to the subfornical organ, one of the sites of Ang II action in the brain. Physiologically, it lacks a blood-brain barrier, so many circulating mediators can activate the ANS centrally.^57^ Immune cells have been shown to express beta-adrenergic receptors. The sympathetic activation stimulated the proliferation of T lymphocytes and dendritic cells and their infiltration into organs and arteries, indicating arteriocerebral junctions.^55^ On the other hand, immune cells reside within the central nervous system and can enhance sympathetic activation. It has been suggested that microglia account for 20% of glial cells and are brain-resident macrophages.^58^ Stimulation of microglia causes polarization of macrophages towards the secretion of pro-inflammatory cytokines IL-1β, TNF-α, and sympathetic stimulation, accelerating hypertension, while blocking microglia using kinin antagonists, TLR4 receptors, or chemically modified tetracycline-3 (CMT-3), reduces neuroinflammation.^59–61^

Despite the significant evidence regarding the activation of the immune system in the pathogenesis of hypertension, attempts to use immunosuppressive drugs in animals or humans are not very promising due to side effects. In the CANTOS study, canakinumab, an anti-IL-1β agent, reduced cardiovascular risk but caused severe infections, and the blood pressure reduction results were insignificant.^62^ The use of tocilizumab (anti-IL-6 antibody) or rituximab (anti-CD20 antibody) reduced hypertension in people with RA and psoriasis, but at the same time increased total cholesterol levels, adversely affecting cardiovascular risk.^63^ One recent meta-analysis showed that many immunosuppressive drugs increase the risk of major cardiovascular events.^64^ This is particularly visible in the case of cyclosporine, which, through dyslipidaemia, endothelial damage, hyperglycaemia, and hyperuricemia, increases CVD despite inhibiting the immune system and increasing hypertension.^65^ This confirms that despite much evidence suggesting the activation of the immune system in the pathogenesis of hypertension, finding ways to inhibit it safely is a significant challenge.

## 3. Heart fibrosis

### 3.1 Activation of fibroblasts

Cardiac fibroblasts (CF) are an essential component of the heart muscle. It is estimated that the myocardium comprises about 75% cardiomyocytes, between which there are layers of ECM. It consists mainly of collagen fibres (80% collagen type I with thicker and durable fibres, 10% collagen type III, and 10% other types of collagen IV, V, and VI) and there are also vascular endothelial cells, pericytes, and small amounts of macrophages and dendritic cells.^66^ Furthermore, it is a storehouse of growth factors and various glycoproteins. Collagen fibres directly surround and reinforce individual cardiomyocytes, forming the endomysium and the outer one layer (perimysium), which group individual cardiomyocytes into bundles and protect against excessive stretching. The outermost collagen fibres forming the epimysium ensure the proper positioning of groups of cardiomyocytes. CF are a source of collagen and metalloproteinases responsible for maintaining constant ECM. CF receive paracrine signals from cardiomyocytes, immune system cells, chemokines, cytokines, mechanical stimuli (e.g. stretching), and the nervous system. When properly stimulated, they can transform into myofibroblasts (MyoFB), producing significant amounts of ECM.^67^ There are several types of fibrosis, including replacement, interstitial, and perivascular fibrosis. Replacement fibrosis occurs after a myocardial infarction (MI) when damaged cardiomyocytes are replaced by ECM, which forms a scar. The interstitial fibrosis is the deposition of excess collagen fibres in the absence of significant loss of cardiomyocytes (cardiomyopathies, hypertension). Perivascular fibrosis includes the accumulation of collagen around the blood vessels. Histologically, four types of collagen fibre deposition can be distinguished—interstitial, dense, patchy, and diffuse.^68^

The origin of MyoFB, which is responsible for excessive ECM deposition, is different. Many of them come from residual fibroblasts and mesenchymal stem cells, but often they originate from other tissues. Under the influence of growth factor stimulation, such as platelet-derived growth factor (PDGF) and fibroblast growth factor (FGF), epithelial and endothelial cells can transform into myofibroblasts in the processes of epithelial to mesenchymal transition (EMT) and endothelial to mesenchymal transition (EndMT).^69,70^ Under the influence of signalling stimuli, endothelial cells lose expression of CD44 and vimentin, characteristic of epithelial cells, and begin to express α-smooth muscle actin (α-SMA), characteristic of MyoFB. It is believed that during overload pressure, heart failure up to 70% of MyoFB arises as a result of EndMT.^71^ Active MyoFB may also originate from pericytes. It is unclear whether bone marrow-derived stem cells (BMSCs) that infiltrate the damaged heart may be the direct source of MyoFB. The majority BMSCs infiltrating injured cardiomyocytes are macrophages and other immune cells, while evidence for other BMSCs subtypes directly giving rise to MyoFB remains limited.^72^ Recently, there have also been reports about the possibility of transforming adipose tissue progenitor cells. Fibro-adipogenic progenitor cells, under the influence of osteopontin (OPN), gained the ability to transform into MyoFB.^73^ Further studies are needed to explain what types of cells and mechanisms can be the source of MyoFB responsible for excessive pathological fibrosis and the possibility of inhibition of EndMT.

### 3.2 Matricellular proteins—recent advances in fibrosis and inflammation

As the ECM is composed of many different cells, it is essential to distinguish between fibroblasts and, among them, resting fibroblasts from active MyoFB. Typical resting fibroblasts express transcription factor 21(Tcf21), discoidin domain receptor 2 (DDR2), and platelet-derived growth factor receptor α (PDGFRα).^74^ The change in activated MyoFB results in the appearance of α-SMA, which is present in most of this group of cells, although some of them, especially in the early stages of differentiation, may not show its expression. Recent studies also indicate periostin (POSTN), fibroblast activator protein (FAP), and tenascin C (TNC) as the markers of active MyoFB. This is a group of ECM glycoproteins involved in fibrosis and increased inflammation. In the models of induced fibrosis, fibroblasts also showed thrombospondin-4 (Thsp4) expression and cartilage intermediate layer protein 1 (Cilp) without expression of α-SMA.^75^ It is difficult to precisely understand the relationship between the expression of individual markers on fibroblasts, mainly since many of them, such as fibronectin, vimentin, fibroblast-specific protein 1 (FSP1), and stem cell antigen-1 (Sca-1), are expressed on many cells that are part of the ECM.^76^

#### 3.2.1 Periostin

ECM glycoproteins strongly expressed on active myofibroblasts show a significant relationship with the activation of the immune system. POSTN has been primarily known for a long time as a chemoattractant, increasing the expression of pro-inflammatory cytokines, especially IL-4 and IL-13. Its plasma concentration has been shown to correlate with the severity of eosinophilic asthma.^77^ Increased POSTN secretion, intensified by IL-1-β and TNF-α, was also observed on fibroblasts during RA, which correlated with joint damage and lung fibrosis.^78^ Recently, it has been shown that in patients with systemic sclerosis (SSc), plasma periostin levels are proportional to disease activity via activation of monocytes to CD86−/CD206+ M2 macrophages and secretion of pro-inflammatory cytokines.^79^ The data on the role of POSTN in the heart have been limited, but recently several studies have been published. POSTN levels, which correlate with the severity of fibrosis, were elevated in patients with heart failure and post-myocardial infarction.^80^ Similar observations occurred in diabetic cardiomyopathy (DCM), diabetic mice, and high-glucose incubated CF, where glucosyringic acid (GA), a periostin inhibitor, ameliorated DCM.^81^ Recent spatial transcriptomics studies have shown that the interaction between the macrophage CCR2 receptor and IL-1β plays a role in the emergence of active cardiac FAP/POSTN fibroblasts. The depletion *in vivo* of IL-1β or macrophage CCR2 ligand correlated with a reduction in myocardial fibrosis and reduced FAP/POSTN fibroblasts.^82^ Moreover, in mice with pressure-overloaded hearts and patients with non-ischaemic dilated cardiomyopathy, scleraxis (Scx), a transcription factor regulating POSTN expression, was overexpressed, and tamoxifen-inducible fibroblast-specific scleraxis knockout (Scx-fKO) completely restored cardiac systolic function.^83^

#### 3.2.2 Tenascin C

TNC is another large group of glycoproteins characteristic of activated myofibroblasts, not present in the normal heart but strongly expressed during embryological development and in fibrotic failure. Its expression typically increases 3–5 days after myocardial infarction.^84^ TNC is strongly associated with immune activation. Tenascin C acts as DAMPs by stimulating the TLR4 receptor, which activates the NLRP3 inflammasome, increases M1 polarization of macrophages and stimulates the expression of pro-inflammatory cytokines through integrins αvβ3 present on macrophages.^85,86^ TLR4 blockade on isolated porcine mitral valve endothelial cells (MVEC) inhibited valve fibrosis and TNC-dependent EMT in pigs and sheep.^87^ Furthermore, TNCs have different chemokine-binding patterns, especially CCL2 and CCL26, whose binding influences eosinophil and monocyte chemotaxis.^88^ TNC also stimulates fibrosis by affecting the TGF-β/Smad2/Smad3 signalling pathway.^89^ Recent published studies show that overexpression of N6-adenosine-methyltransferase METTL-3, which causes an increase in N6-methyladenosine (m^6^A) mRNA encoding TNC and increases its stability, causes increased cardiomyocyte apoptosis and progressive fibrosis. At the same time, the m^6^A mutation and reduced TNC expression resulted in a cardioprotective effect.^90^ Similar effects were achieved by silencing the transcription factor Prrx1, which stimulates TNC expression in the TGFβ-dependent Twist1-Prrx1-TNC-PFL signalling cascade.^91,92^ Analysis of 200 patients with advanced heart failure showed that elevated plasma TNC levels were associated with increased 1-year mortality, similar to NT-proBNP and hs-CRP.^93^ In another observational study, plasma TNC levels additionally correlated with clinical severity of heart failure (NYHA scale), inflammatory parameters (myeloperoxidase, IL-6, TNFR-1-tumour necrosis factor 1, the mediator of TNFα), and fibrotic parameters (Galectin-3, metalloproteinases, and tissue inhibitors of metalloproteinases TIMP-1, TIMP-4).^94,95^

#### 3.2.3 Thrombospondins

Another group of active ECM proteins includes thrombospondins (Thsp), which participate in myocardial remodelling, especially in chronic kidney disease (CKD). In the course of CKD, the expression of the Thsp1 receptor in the myocardium increases and mice lacking Thsp1 are protected against hypertrophy and fibrosis.^96^ Thsp, similarly to POSTN and TCN, stimulates TGF-β. It has been shown that the use of long noncoding RNA termed thrombospondin 1 antisense 1 (Thsp1-AS1) reduces the activation of CF by TGF-β, similarly to the use of TGF-β pathway antagonists, which inhibit the profibrotic effect of Thsp1.^97^ It was also demonstrated that the application of lipopolysaccharide (LPS) to mouse cardiomyocytes increased the amount of Thsp1 mRNA, and its silencing decreased the levels of pro-BNP, cTnI, TNF-α, IL-6, and caspase-3, which reduced cardiomyocyte apoptosis. Plasma Thsp1 levels significantly increased in patients with sepsis, causing myocardial damage, suggesting the importance of inflammation and the role of Thsp1.^98^ Furthermore, thrombospondin 2 was identified as a protein biomarker of early-onset heart failure based on data analysis from three cohorts—HOMAGE, Aric, and FHS.^99^

#### 3.2.4 Osteopontin

Osteopontin (OPN), also known as secreted phosphoprotein 1 (Spp1), is also worth mentioning. This glycoprotein produced mainly by macrophages, acts as a pro-inflammatory cytokine and an ECM protein. OPN is more highly expressed in cardiomyopathies and heart and kidney failure, accompanied by infiltration of Spp1 macrophages in these organs.^100,101^ Recent publications have shown that one of the main factors stimulating Spp1 macrophages to secrete profibrotic factors are chemokines and their receptors, particularly CXCR4 secreted by activated platelets due to injury. Loss of CXCR4 inhibits Spp1 macrophage differentiation, which was associated with a reduction in cardiac and renal fibrosis.^102^ Moreover, Spp1 macrophages, which mainly inhabit perivascular adipose tissue (PVAT), are considered one of the main factors of perivascular fibrosis. OPN secreted by Spp1 macrophages binds with CD44 and integrin on fibro-adipogenic progenitor cells, stimulating their division and differentiation and enhancing coronary PVAT fibrosis.^73^ This is one of the first studies showing that adipocyte precursor cells can be a source of active myofibroblasts. This finding is exciting in the context of a recent publication indicating increased infiltration of monocytes and Spp1 macrophages in human atria in atrial fibrillation, the deletion of which reduced the severity of the arrhythmia.^103^ OPN may be one of the factors responsible for the development of heart failure in HIV. OPN transcripts were increased in mouse embryonic fibroblasts stimulated with LPS. In a mouse model of HIV infection, increased OPN concentrations were observed in plasma and cardiac ventricles. This was associated with increased infiltration of activated CD14 + CD16+ monocytes and increased fibrosis.^104^

All the above matrix cellular proteins have been known for a long time, and new data on their activation, stimulation, and interaction with other cells are constantly emerging. Due to their expression in many cells and organs and their fulfillment of many functions in molecular signal transmission, it is not possible to completely block them. However, it is essential to learn the details of activation in individual organs and various pathological conditions to determine the particles and compounds that participate in signal transmission and their activation. These particles could be the target of therapy, protecting against further activation of fibroblasts and progression of inflammation in the fibrotic heart. *Table 1* summarizes the main ECM proteins of MyoFB, their biological activity, and their importance in molecular immune activation and fibrosis.

**Table 1:** Main ECM proteins of MyoFB with molecular mechanisms of immune activation and fibrosis

ECM protein	Biological activity	Molecular mechanisms of immune system activation and fibrosis	Human/animal research
Periostin (POSTN)	FAS1 glycoprotein Ligand of αVβ_3_ and αVβ_5_ integrin	↑FAP/POSTN fibroblasts by interaction with macrophage CCR2 receptor and IL-1β^82^ ↑Differentation of monocytes into CD86−/CD206 macrophages^79^ ↑Expression IL-4, IL-13^77^ ↑TGF-β signalling^105^	↑POSTN plasma levels in patients with acute MI, correlated negatively with EF and positively with left ventricular end-diastolic diameter^80^ ↑expression of Scx in mice pressure-overloaded hearts^83^ ↓DCM activity in diabetic mice and *in vitro* on CF after GA administration, a POSTN inhibitor^81^ ↑POSTN expression on fibroblasts after IL-1β and TNF-α stimulation in RA^78^ ↑POSTN plasma levels correlated with Ssc disease activity^79^
Tenascin C (TNC)	Hexameric glycoprotein highly expressed during embryogenesis and after tissue injuries	↑TLR4, NLRP3 activation (DAMPs activity)^85^ ↑M1 polarization of macrophages^85^ ↑Eosinophils and monocytes chemotaxis by CCL2/CCL26 interaction^88^ ↑METTL3 expression^90^ ↑TGF-β stimulation^89^	↑TLR4 blockade in pigs and sheep inhibited TNC-dependent EMT^87^ ↑TNC levels correlated with increased 1-year mortality in patients with HF and NYHA scale^93^ ↑TNC levels increase plasma concentration of IL-6, TNF-α, galectin-3 and metalloproteinases^94^
Thrombospondins (Thsp)	Homotrimeric glycoprotein with three properdin-like repeats, Thsp1 and Thsp2 family	↑TGF-β stimulation (TGF−β antagonist inhibits profibrotic effect of Thsp1)^97^	↑Thsp1 plasma concentration in patients with sepsis and myocardial damage^98^ ↓Thsp1 mRNA in mouse cardiomyocytes decreased level of NT-proBNP, TNF-α, IL-6^98^ Mice lacking Thsp1 receptors in myocardium are protected against hypertrophy and fibrosis^96^
Osteopontin (OPN)	Acidic glycoprotein, pro-inflammatory cytokine, produced mainly by macrophages	↑OPN secretion after CXCR4 stimulation of macrophages^102^ ↑Binding to CD44 and integrins on FAP^73^ ↑Activation CD14 + CD16+ monocytes^104^	↑Infiltration of OPN macrophages in cardiac tissue in patients with HF^100^ ↑OPN macrophages in human atria in atrial fibrillation^103^ ↑OPN concentration in cardiac tissue and plasma in mice with HIV^104^

MyoFB produces excessive amounts of ECM, impairing cardiomyocyte contractility and increasing oxygen demand. MyoFB expresses several characteristic matricellular proteins such as periostin, tenascin C, thrombospondins and osteopontin, which, by using immune stimulation, activate TGF-β-dependent profibrotic signalling pathways.

FAP, fibroblast activation protein; FAS1, fasciclin one domain; Scx, scleraxis; DCM, diabetic cardiomyopathy; GA, glucosyringic acid; RA, rheumatoid arthritis; Ssc, systemic sclerosis; EMT, epithelial to mesenchymal transition.

### 3.3 TGF-β—main profibrotic factor

Matricellular proteins (POSTN, TNC, Thsp and OPN), integrins, and proteasomes activate TGF-β signalling. TGF-β is the main factor responsible for fibroblast stimulation and fibrosis, the most thoroughly studied in this area. Many profibrotic molecules and factors affect the TGF-β signalling cascade. TGF-β occurs in three isoforms (TGF-β 1, 2, 3) that act on the same receptor group but with different sensitivity, affinity, and regulation patterns.^106^ Latent TGF-β activated by ECM proteins stimulates the ALK5-related receptor and triggers a canonical Smad-dependent and non-canonical (Smad-independent) signalling cascade that promotes the expression of profibrotic genes in the cell nucleus. The canonical cascade is associated with Smad2/Smad3/Smad4 phosphorylation. At the same time, the non-canonical pathway involves phosphatidylinositol kinase (PI3K), p38 mitogen-activating protein kinase (p38 MAPK), c-Jun N-terminal kinase (JNK), extracellular signal-regulated kinase (Erk1/2), Rho family of GTPases, and also JAK/STAT and Wnt/β-catenin signalling pathway.^107^ Numerous studies in mice have shown that overexpression of TGF-β leads to increased fibrosis and production of ECM proteins, while deletion reduces intestinal fibrotic changes and heart remodelling.^108^ Other factors causing fibrosis, such as radiotherapy, anthracycline therapy, arrhythmias, and cardiomyopathies, are also associated with the action of TGF-β, so research is ongoing to find a molecule that could inhibit this transmission.^109,110^

However, despite the significant benefits of inhibiting TGF-β signalling in terms of myocardial fibrosis remodelling, treatment in experimental models was associated with a substantial risk of adverse events related to the physiological effects of TGF-β. Complete blockade of TGF-β transmission (e.g. using fresolizumab (an antibody against TGF-β), despite reducing fibrosis, significantly increased inflammation, destroyed the structure of coronary arteries, increased mortality when administered shortly after myocardial infarction and caused extracardiac changes such as the formation of keratoacanthomas.^111,112^ ALK5 antagonists (galunisertib, GW788388, SD-208, SM-16) in mice, despite reducing post-infarction left ventricular systolic function and fibrosis, caused valvular degeneration and ventricular dilatation.^113,114^ Genetic deletion of Smad2 or Smad3 increased the risk of post-infarction ruptures, indicating their essential role in structural integrity.^115,116^

For this reason, blocking single molecular factors affecting TGF-β or mechanisms that only partially eliminate the action of TGF-β seems to be the most appropriate way to find an effective drug in reducing fibrosis. Recent studies indicate a specific role of integrins αVβ1 and αVβ6 on fibroblasts, which cooperate in the release of mature TGF-β. There are currently few molecules that effectively block them, such as cilengitide, an antagonist of the αV integrin subunit, which reduced fibrosis and inflammatory infiltration and post-infarction mortality in mice.^117^ New integrin-blocking molecules are on the horizon but have not yet been tested in cardiac fibrosis.^118^ Another interesting option is pentosan polysulphate, an inhibitor of ADAMTS4 (A disintegrin and metalloprotease with thrombospondin Motif 4), which, together with Thsp1, increases the bioavailability of TGF-β. In studies on mice, it reduced fibrosis and heart failure, which was also associated with its good tolerance.^119^ Recent studies also point to a latency-associated peptide (LAP) responsible for TGF-β activation and facilitating its binding to the receptor. Blocking LAP on mouse CF reduced inflammation and fibrosis and reduced the level of integrins avβ3, αvβ5 and p-Smad2, and p-Smad3.^120^

Additionally, it has been shown that TGF-β signalling is critically important in vascular homeostasis, playing a key role in smooth muscle cells. Proteins regulating the process of TGF-β maturation from LAP-TGFβ, including Emilin-1, were involved in hypertensive vascular remodelling.^121^ In particular, the ablation of Emilin-1 in smooth muscle cells determined an increased availability of activated TGF-β, which in turn promoted increased contractility of resistance arteries, overall contributing to blood pressure increase.^122,123^ On the other hand, the selective inhibition of TGF-β signalling in smooth muscle cells, obtained by deleting Smad4, provoked alterations in the elastic lamellae and extracellular matrix, lastly leading to aneurysm formation.^124^ Taken together, these observations indicate that the complexity of TGF-β in the homeostasis of the cardiovascular system requires further investigation.

These data support the conclusion that the best target for the development of antifibrotic therapies related to the TGF-β pathway should be based on the activation of TGF-β and interaction with ECM, integrins, and smaller molecules, which increases the chances of avoiding adverse effects resulting from complete TGF-β blockade.

### 3.4 The RAA system—from hypertension through inflammation to fibrosis

The RAA system, whose activation is a fundamental mechanism in developing hypertension, also plays a vital role in the fibrosis process. More than 20 years ago, it was shown that stressed cardiomyocytes and fibroblasts can produce renin and angiotensin-converting enzyme (ACE) that converts renin to angiotensin.^125^ Furthermore, cardiomyocytes and fibroblasts express on their surface angiotensin receptors, especially AT1. Stimulation of the AT1 receptor via activation of p38MAPK, Erk cascades, and protein kinase C (PKC) leads to the activation of myofibroblasts and integrins and the stimulation of fibrosis.^126^ Since it shows similarity to the non-canonical pathway of TGF-β action, TGF-β is an important co-stimulant of the profibrotic action of Ang II.^127^ In turn, AT2 receptor stimulation has the opposite effect of reducing myocardial remodelling and inflammation. Unfortunately, it has been observed that its expression decreases after myocardial infarction or damage, and the AT1/AT2 ratio increases.^128^ So far, there are not many AT2 agonists available. Recently, β-substituted angiotensin III peptide (β-Pro7-AngIII) was tested on mice, which, after 4 weeks of treatment, reduced myocardial fibrosis, and reduced local and systemic TGF-β concentration and inflammation.^129^ Edaravone, an antioxidant compound currently of broad interest in neurology, increased AT2 expression in the myocardium at the AT1 receptor stripe and correlated with a reduction in myofibroblast activity and macrophage infiltration.^130^ Due to the currently limited ability to influence AT2, it focuses mainly on the efficient blocking of AT1 and inhibition of ACE and neprilysin, which break down Ang II and natriuretic peptides.^131^ AT1 receptors are a G protein-coupled family dependent, and their profibrotic effects are mediated by Gαq-dependent and β-arrestin-independent subunits, which act on TGF-β and endothelin 1 (ET-1). Simultaneous blockade of TGF-β and ET-1 completely abolishes Ang II-dependent stimulation of myofibroblasts.^132,133^ Moreover, AT1 stimulation was found to be associated with Hippo-yes-associated protein (YAP), a mechanoreceptor protein highly expressed in the stiff matrix, the blocking of which significantly reduced CF proliferation and TGF-β activity.^134–136^ This allows us to conclude why fibrotic and stiff myocardium undergo further fibrosis, regardless of other profibrotic factors. A unique role in the studies is given to sacubitril/valsartan (Sac/Val), a neprilysin inhibitor registered for treating chronic heart failure. It has been shown that Sac/Val reduces fibroblast proliferation by inhibiting p-Smad2/3, p38 MAPK, and p-JNK (like TGF-β)^137^ and restoring protein kinase G (PKG), which inhibits the activation of Rho, involved in the activation of myofibroblasts.^138^ Furthermore, its action on cGMP/PKG reduces the phosphorylation of titin, a large sarcomeric protein responsible for the stiffness of the myocardium.^139^ In mice subjected to aortic banding, Sac/Val inhibited the activation of NLRP3 inflammasome and NF-κB.^140^ Moreover, it attenuated the M2 polarization of macrophages, myocardial infiltration of CD206+ macrophages, epithelial to mesenchymal transition, and expression of profibrotic factors.^141,142^ The anti-inflammatory effect of Sac/Val may also be mediated by the tryptophan/kynurenine pathway. In rats with heart failure, it reduced the expression of indoleamine 2,3-dioxygenase (IDO), where, in addition to the decrease in plasma NT-proBNP and collagen concentration in the myocardium, a reduction in the myocardial expression of IL-1β, TNF-α, IL-6, or IFN-γ was observed.^143,144^

**Figure 2:**
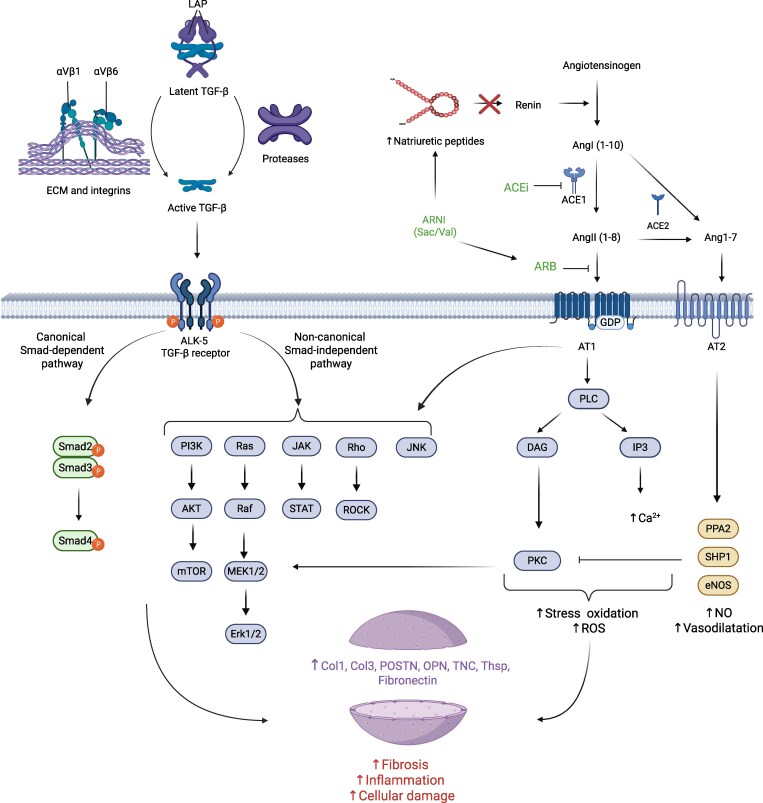
TGF-β and Ang II signalling cascade activate profibrotic pathways involved in the increased expression of ECM proteins of MyoFB. TGF-β is released from its inactive form associated with LAP due to interaction with integrins and the activity of metalloproteinases, and acting on the ALK-related receptor triggers canonical Smad-dependent and non-canonical Smad-independent signalling pathways responsible for heart fibrosis. Angiotensin II acting on the AT1 receptor influences many Smad-independent signalling pathways dependent on TGF-β. This explains the antifibrotic effect of commonly used RAA inhibitors. This figure has been created with Biorender.com. LAP, latency-associated peptide; Sac/Val, sacubitril/valsartan; ECM, extracellular matrix; ACE, angiotensin-converting enzyme; ACEi, angiotensin-converting enzyme inhibitors; ARNI, angiotensin receptor-neprilysin inhibitor; ROS, reactive oxygen species; Col1, Col3, types of collagen; POSTN, periostin; OPN, osteopontin; TNC, tenascin C; Thsp, thrombospondin; TGF-β, transforming growth factor beta; PLC, phospholipase C; DAG, diacylglycerol; IP3, inositol trisphosphate; PKC, protein kinase C; PPA2, inorganic pyrophosphatase 2; SHP1, protein tyrosine phosphatase with SH2 domain; eNOS, endothelial nitric oxide synthase; JNK, c-Jun N-terminal kinase; Erk1/2, extracellular signal-regulated kinase; PI3K, phosphatidylinositol kinase; mTOR, mammalian target of rapamycin; ROCK, Rho-associated coiled-coil kinase.

This novel evidence explains why RAA inhibitors, especially ARNI, show such practical and pleiotropic effects in treating hypertension and HF. *Figure 2* presents the molecular mechanisms of their action, together with the TGF-β signalling cascade.

### 3.5 Cytokines and chemokines in heart fibrosis

As mentioned above, cytokines and chemokines play an essential role in the pathogenesis of hypertension and accompany the activation of myofibroblasts. Among the 10 genes most involved in cardiac fibrosis in the Gene Expression Omnibus (GEO) cohorts RNA sequencing, as many as 6 of them (IL-6, Il-1β, TLR4, IL-10, CCL2, CXCL8) were closely related to the immune system.^145^ IL-1β has long been known as a pro-inflammatory and profibrotic cytokine that transforms fibroblasts into myofibroblasts, activating MMPs and blocking MMP inhibitors.^146^ In addition, it stimulates the inflammatory process and plaque instability in the pathogenesis of atherosclerosis.^147^ In mice lacking IL-1β, reduced collagen deposition in the myocardium and myocardial remodelling after myocardial infarction were observed.^148,149^ Similar effects are demonstrated by IL-6, which stimulates STAT3 and TGF-β.^150^ IL-1β and IL-6 are the central cytokines produced upon activation of the NLRP3 inflammasome, and their expression is linked to the stimulation of the TLR4 receptor in the TLR4/NF-κB/NLRP3 signalling pathway. Molecules that could block the TLR4 and NLRP3 pathways are being tested experimentally and have shown significant benefits in cardiac function.^151^ Artesunate (an antimalarial drug) reduces fibrosis and cardiac hypertrophy by inhibiting myeloid differentiation factor 2 (MD2), acting on the TLR4 receptor.^152^ Darapladib, a lipoprotein-associated phospholipase A2 (Lp-PLA2) inhibitor, a drug in clinical trials for the treatment of atherosclerosis, reduces the expression of NLRP3 and IL-1β and the recruitment of macrophages in the myocardium by inhibiting the stimulation of fibroblasts.^153^ Phloretin and lupeol, anti-inflammatory cosmetic preparations, reduced myocardial remodelling via NLRP3/Caspase-1/IL-1β and TLR4-PI3K-Akt-NF-κB in rats and mice, respectively.^154,155^ Genetic depletion of NLRP3 also had a strong cardioprotective effect.^156^

Recently, attention has been drawn to IL-33, a member of the IL-1 family and IL-11 from the IL-6 family. IL-33 acts by inhibiting the tumorigenicity 2 receptor (sST2). The IL-33/ST2 axis has been considered an antiproliferative and anti-inflammatory pathway that limits the action of sST2, a marker of HF. Some studies confirm that IL-33 exerts a cardioprotective effect on the myocardium, and its ablation exacerbates HF.^157^ Nevertheless, the role of IL-33 is ambiguous. In other organs, such as the liver or lungs, it has a strong pro-inflammatory and pro-fibrotic effect. IL-33 activates Th2 and TcCD8+ lymphocytes and increases the secretion of IL-17A.^158^ Recently, in studies in Zucker fatty rats, overexpression of IL-33/ST2 led to increased fibrosis in adipose tissue and myocardium. In humans with HF, it correlated with the expression of CTGF and TGF-β1.^159,160^ Yap/taz, a myocardial stiffness mechanoreceptor, is also involved in IL-33 expression. Neutralizing IL-33 inhibited its profibrotic effect.^161^ IL-33 also plays a vital role in the stimulation of eosinophil infiltration into the pericardium and the development of pericarditis.^162^ At this stage of knowledge, it can be stated that IL-33 has an anti-fibrotic effect by neutralizing the action of sST2. Still, there are mechanisms, especially in damaged myocardium, that regulate its proliferative and pro-inflammatory action, which further studies should explain.

The situation is similar to IL-11, which has been shown to have cardioprotective effects, especially in the first studies. However, a recent systematic review of eight studies on the role of IL-11 in an animal fibrosis model confirms the strong profibrotic effect of this interleukin.^163^ Expression of IL-11 and its receptor IL-11RA was associated with cardiac hypertrophy in mice subjected to aortic banding, the blockade of which resulted in decreased Erk1/2 transmission and reduced fibrosis.^164^ Furthermore, IL-11/Erk1/2, by decreasing LKB1/AMPK signalling, and the activation of mTOR cassette, stimulates myofibroblasts to produce α-SMA^165^ and TGF-β stimulation leads to an increase in IL-11 in cardiomyocytes. Moreover, IL-11 stimulates the expression of other pro-inflammatory cytokines and IL-33.^166^ Transcriptomics studies have shown that macrophages infiltrating cardiac tissue in the Ang-II-induced hypertension model are characterized by increased expression of methyladenosine demethylases, ALKBH5, which, by demethylating IL-11 mRNA, increases its expression and enhances EndMT and cardiac dysfunction. Blocking this cascade by ALKBH5/IL-11R siRNA administration protects against fibroblast activation and progressive fibrosis.^167^ IL-11 administration to mice caused acute left ventricular damage due to increased activation of NF-κB, TNF-α, and the JAK/STAT3 pathway. Ruxolitinib or tofacitinib showed cardioprotective effects during IL-11 treatment.^168^ There are not many studies yet showing IL-11 levels in CVD, but elevated IL-11 levels have been shown to correlate with plasma and cardiac BNP levels, N-terminal propeptide of type III procollagen (PIIIP) and TGF-β1 in patients with atrial fibrillation.^169^

IL-10 is a significant anti-inflammatory cytokine that acts primarily in an anti-fibrotic manner, although some studies have indicated its stimulatory effect on fibroblasts.^170^ Research involving mice and pigs subjected to acute myocardial infarction has demonstrated that stimulating the IL-10 receptor, through the STAT-3 signalling cascade, which inhibits the pro-inflammatory transcription factor NF-ĸB, has a cardioprotective effect. In contrast, mice lacking the IL-10 receptor exhibited more extensive inflammatory cell infiltration and increased fibrosis.^171^ In animal models, a hydrogel releasing IL-10 and SN50 (an NF-κB blocker) is being tested, which, through IL-10's anti-inflammatory effect, improves healing at the infarct site.^172^ The IL-10/STAT3 pathway also contributes to the myocardial infiltration of myeloid-derived suppressor cells (MDSCs), potent immunosuppressive cells, the numbers of which have been noted to increase in mice subjected to exercise training.^173^ Furthermore, it has been shown that the cardioprotective effect of Treg lymphocytes relies on IL-10 action. Tregs deprived of IL-10 lost their ability to neutralize TcCD8+ lymphocytes and activated macrophages.^174^ Human amniotic mesenchymal stem cells (hAMSCs) administered to mice with stimulated fibrosis and inflammation, which upregulate IL-10 expression, resulted in significant cardioprotection through increased polarization of CD206 + IL10+ macrophage infiltration and decreased expression of α-SMA and collagens.^175^ Additionally, it is important to note that IL-10 inhibits the infiltration of bone marrow fibroblast progenitor cells into the myocardium and their transformation into myofibroblasts.^176^

Chemokine CCL2, or monocyte chemotactic protein 1 (MCP-1), is a C–C motif chemokine family cytokine. It is one of the primary factors stimulating the infiltration of other inflammatory cells by binding to the CCR2 receptor. It was observed that 24 h after ligation of the left coronary artery and subsequent reperfusion in mice, there was a peak of CCL2 secretion by stimulated macrophages. CCL2/CCR2 activated the NLRP3/Caspase-1/IL-1β axis in the heart, influencing the infiltration of profibrotic M2 macrophages and increasing TGF-β and expression of Col1α1 in fibroblasts. Administration of the CCR2 inhibitor to mice or CCR2 gene knockout (CCR2^−/−^mice) reduced inflammation and TGF-β levels. This was correlated with reduced MI area, cardiomyocyte damage, and improved heart function.^177^ Decreased CCL2 expression correlated with reduced p38/MAPK/NF-κB pathway activity and pro-inflammatory M1 macrophage infiltration in the infarcted area.

Furthermore, LPS-induced TNF-α secretion by M1 macrophages was significantly reduced in CCL2 deficiency. In turn, CCL2 overexpression attenuated the action of the anti-inflammatory cytokine IL-10 by M2 macrophages.^178^ Patients with chronic heart failure (CHF) compared to non-CHF showed higher plasma (MCP)-1/CCL2 levels, which correlated with their concentration in the bronchoalveolar lavage and the severity of lung fibrosis, as a complication of CHF-increasing dyspnoea.^179^

The role of chemokines from the C-X-C motif chemokine family, especially CXCL4, in the pathogenesis of cardiac fibrosis has also been confirmed. As mentioned above, CXCL4, through the CXCR4 receptor, stimulates the infiltration of Spp1 macrophages and is responsible for the profibrotic effect of OPN.^102^  *In vitro*, CXCL4 has been shown to stimulate EndMT and differentiation of myofibroblasts, including epithelial cells.^180^ Additionally, in the model of severe myocarditis in mice, activation of the profibrotic Wnt/β-catenin pathway increased the secretion of CXCL4, which intensified fibrosis by stimulating the PI3K/AKT pathway.^181^

This evidence highlights the critical role of cytokines, chemokines, and their receptors in the inflammatory process and progressive fibrosis, especially in activating macrophages and interacting with matricellular proteins. *Table 2* summarizes the exact role of individual cytokines and chemokines.

**Table 2:** The function of the essential cytokines and chemokines in the molecular activation of profibrotic cascades and the development of cardiac fibrosis

Cytokine/chemokine	Biological activity	Molecular mechanisms of immune system activation and fibrosis	Human/animal research
IL-1β/IL-6	Leukocytic pyrogen, strong pro-inflammatory cytokines, secreted through TLR4/NLRP3/caspase-1 activation	↑MMPs^146^ ↓TIMPs^146^ ↑STAT3^150^ ↑TGF-β signalling^150^ ↑NF-κB^154,155^	↓TLR4 signalling by artesunate, reduced fibrosis associated gene expression in iPSC-CF—human induced pluripotent stem cell^152^ ↓NLRP3/caspase-1/IL-1β by phloretin inhibits inflammation, collagen expression and cardiac impairments in rats^154^ ↓ANP, BNP and inflammatory markers in TAC mice heart tissue after lupeol treatment^155^ ↓Cardiac hypertrophy after NLRP3 genetic depletion in mice^156^
IL-33	IL-1 family, activation of Th2 lymphocytes and mast cells	↓ST2^157^ ↑Th2 lymphocytes^158^ ↑Tc CD8+ lymphocytes ^158^ ↑IL-17A^158^ ↑Yap/taz mechanoreceptor^161^	IL-33^−/−^ TAC mice presented higher cardiac hypertrophy and impaired cardiac function^157^ ↑IL-33/ST2 expression in rats increased fibrosis^159^ ↑ TGF-β1 and CTGF in patients with HF^159,160^
IL-11	IL-6 family, produced by osteoblasts and bone marrow stromal cells, stimulates thrombocytopoiesis	↑Erk1/2 transmission^164^ ↓ LKB1/AMPK signalling^165^ ↑ mTOR axis^165^ ↑NF-κB^168^ ↑TNF-α^168^ ↑JAK/STAT3^168^ ↑IL-33 and pro-inflammatory cytokines^166^	↑IL-11/IL-11R correlated with cardiac hypertrophy in mice after aortic banding^164^ ↓IL-11 activity in cardiac macrophages inhibited fibroblast activation^167^ ↑Left ventricular damage in mice after IL-11 administration^168^ ↑IL-11 plasma levels correlated with increased BNP, PIIIP and TGF-β1 levels in patients with AF^169^
IL-10	cytokine synthesis inhibitory factor, inhibits the secretion of TNF-α, IFN-γ, GM-CSF	↑STAT3^171^ ↓NF-κB^172^ ↑ MDSCs cardiac infiltration^173^ ↓ BMSCs cardiac infiltration^176^ ↑Treg activity^174^ ↑ CD206 + IL10+ macrophage polarization^175^	↑CF and inflammation after acute MI in mice without IL-10 receptor^171^ ↑Healing the infarct size in mice by using hydrogel releasing IL-10^172^ ↓α-SMA and collagens expression in mice with IL-10 upregulation^175^
CCL2 (MCP1)	C-C motif chemokines family, monocyte chemotactic protein	↑NLRP3/caspase-1/IL-1β^177^ ↑TGF-β signalling^177^ ↑Col1α1 expression^177^ ↑ p38/MAPK/NF-κB^178^ ↑ M1 infiltration^178^ ↑TNF-α^178^ ↓IL-10^178^	↑CCL2 macrophages secretion in TAC-mice after reperfusion^177^ ↓TGF-β levels and reduction MI area in CCR2^−/−^mice and after CCL2 inhibitor administration^177^ ↑CCL2 concentration in plasma and BAL in CHF patients^179^
CXCR4	C-X-C motif chemokine family, activating macrophages	↑Spp1+ macrophages infiltration in myocardium^102^ ↑ PI3K/AKT signalling^181^	↑EndMT and MyoFB differentiation^180^ ↑Cardiac inflammation and fibrosis in severe myocarditis model in mice^181^

In the course of heart failure, it was observed increased expression of strongly pro-inflammatory cytokines from the IL-1β and IL-6 family dependent on the activation of the NLRP3 inflammasome, including IL-33 and IL-11, as well as chemokines, especially CCL2 and CXCR4. They further intensify inflammation and activate profibrotic signals dependent on TGF-β. Furthermore, anti-inflammatory IL-10, inhibiting profibrotic signals, is reduced after myocardial infarction and during oxidative stress.

MMPs, matrix metalloproteinases; TIMPs, tissue inhibitor of metalloproteinases; iPSC-CF, human induced pluripotent stem cell-derived cardiac fibroblasts; CTGF, connective tissue growth factor; AF, atrial fibrillation; MDSCs, myeloid-derived suppressor cells; BMSCs, bone marrow-derived stem cells; EndMT, endothelial to mesenchymal transition; CHF, chronic heart failure; Spp1, secreted phosphoprotein 1; TAC, transverse aortic constriction.

## 4. Neuromodulation—dysregulation of the autonomic nervous system in heart fibrosis and inflammation

During heart failure and fibrosis, dysregulation of the autonomic nervous system (ANS) occurs, characterized by dominant sympathetic activity and reduced parasympathetic stimulation, similar to that frequently observed in hypertension.^182^ The role of adrenergic receptor stimulation in activating cardiac fibrosis has long been recognized, with isoproterenol (ISO), a β-AR agonist, and phenylephrine (PHE), an α2-AR agonist, being used to induce cardiac fibrosis in experimental animal models.^183,184^ Conversely, stimulating β-AR in cardiomyocytes (particularly β1 and β2) is crucial for maintaining their contractile capacity and force. However, prolonged excessive sympathetic stimulation leads to desensitization of β-AR and downregulation of its signalling cascade in cardiomyocytes, ultimately resulting in end-stage heart failure.^185^ Therefore, β-AR blockers have been employed to mitigate the effects of excessive sympathetic stimulation on the heart. Nonetheless, many mechanisms of β-AR signalling on fibroblasts and immune cells remain unknown. Recent publications exploring the integration of adrenergic receptor signals with inflammatory stimuli present an intriguing perspective.

### 4.1 Inflammation and sympathetic hyperactivity

Acute stimulation of α1-AR and β-AR in mice has increased the expression of various pro-inflammatory genes. In particular, activation of α1-AR has enhanced neutrophil infiltration into the myocardium, activated NF-κB, and promoted the production of chemokines.^186^ PHE-stimulated mice exhibited significant myocardial inflammation, activation of the NLRP3 inflammasome, and production of caspase 1 and IL-18.^187^ Additionally, single-cell transcriptomics demonstrate α1-AR expression on macrophages and their stimulation activates fibroblasts to produce collagen.^188^ The activation of β-AR (especially β1AR, which predominates in the heart), as a G protein-coupled receptor (GPCR), stimulates adenylyl cyclase and, through cAMP/PKA and β-arrestin/CaMKII leads to cardiac fibrosis and hypertrophy. The MD2, an activator of the TLR4 receptor, also activates the AR-cAMP-PKA pathway.^189^ In a study on ISO-induced HF mice, AMPK phosphorylated β-arrestin and, by increasing the expression of phosphodiesterase 4 (PDE4), inhibited cAMP/PKA-dependent β-AR stimulation, consequently leading to inhibition of the NLRP3 inflammasome and reduced fibrosis. Similar results were observed following the administration of metformin, an AMPK activator, which reduced cAMP/PKA transmission.^190^ It was also noted that increased AMPK activation occurs during exercise-induced sympathetic stimulation, altering the previously unfavourable sympathetic overactivation.^191^ The results of these studies indicate comparable effects of metformin and physical exercise on the cardioprotective regulation of β-AR transmission by AMPK. Activation of AMPK/NF-κB is also supported by neuregulin 4, an adipokine, which in ISO HF mice significantly limited cardiac fibrosis and hypertrophy while simultaneously decreasing TNF-α, IL-1β, and the apoptotic proteins Caspase-3, Bcl-2, and Bax.^192^

Sympathetic stimulation activates the NLRP3 inflammasome by releasing ATP from sympathetic nerve endings, which acts on P2X purinoceptor 7 (P2X7). Nerve ablation reduces the concentrations of ATP and IL-1β, thereby decreasing cardiac hypertrophy in a mouse model of induced hypertension.^156^ P2X7 is also a potential modulator of electroacupuncture (EA), which attenuates fibrosis in ISO-induced fibrosis mice by reducing P2X7 expression and NLRP3 activation, leading to decreased plasma levels of IL-1β, IL-6, and IL-18. P2X7 knockout mice do not show benefits from EA.^193^ Additionally, IL-18 released by the activated NLRP3 inflammasome in mice correlates with serum norepinephrine concentrations and is a key factor in triggering macrophage infiltration into the myocardium during β-adrenergic stimulation.^194^ Furthermore, IL-6 from the NLRP3 inflammasome activates the profibrotic JAK/STAT3 cascade dependent on beta-adrenergic stimulation.^195^ The Janus kinase inhibitors baricitinib and zanubrutinib, used in ISO-induced fibrosis mice, inhibit the inflammatory process and cardiac remodelling by simultaneously blocking NF-κB, JAK1/2/STAT3, and PI3K/Akt signalling.^196,197^ STAT3 activation resulting from long-term beta-adrenergic stimulation also triggers the activation of PERK, a kinase related to endoplasmic reticulum stress, which disrupts the unfolded protein response through the PERK/eIF2α/ATF4/CHOP pathway, ultimately leading to cardiomyocyte apoptosis.^195^

Furthermore, stimulation of the sympathetic nervous system in the ventrolateral part of the ventromedial hypothalamus (VMHVL), hypothalamic paraventricular nucleus (PVN) and superior cervical ganglion (SCG) of mice is associated with a more substantial infiltration of activated macrophages into the myocardium and post-infarction remodelling and their denervation attenuates these changes.^198,199^ One of the main factors contributing to sympathetic activation in the PVN is TNF-α. It was found that TNF-α acts on EGFR and, stimulates Erk1/2, enhancing the sympathetic activation of the PVN.^200^ Intraventricular infusion of IL-1β and Ang II increased the expression of TNF-α-converting enzyme (TACE), the enzyme responsible for TNF-α production, while reduced TACE mitigated sympathetic stimulation, leading to a significant improvement in the control of hypertension and cardiac fibrosis in HF rats.^201^ These data underscore the importance of neuroinflammation in sympathetic activation and demonstrate a mutual interdependence—sympathetic stimulation induces NLRP3 and the production of pro-inflammatory cytokines, further enhancing sympathetic stimulation.

### 4.2 Cholinergic stimulation—anti-inflammatory and antifibrotic effect?

A recent paper shows that stimulating the caudal nucleus of the solitary tract (cNST) in mice, a nucleus of the parasympathetic nervous system that mitigates the peripheral inflammation process, increases the concentration of the anti-inflammatory cytokine IL-10 while decreasing pro-inflammatory cytokines, which in turn stimulates the vagus nerve and parasympathetic areas of the brain.^202^ These findings complement and confirm the existence of the brain-body axis in the context of the previously less documented anti-inflammatory effect of neurostimulation. One of the most widely described cholinergic receptors in inflammation modulation is the α7nAChR. It is expressed by macrophages, influencing the production of pro-inflammatory cytokines and their activation through modulation of the NF-κB and JAK2/STAT3 pathways.^203^ Moreover, macrophages lacking α7nAChR showed a reduced ability to undergo diapedesis and penetrate inflamed tissues, as well as a diminished binding to ECM proteins due to reduced expression of integrins. Endotoxemia-deficient mice lacking α7nAChR were characterized by increased mortality due to impaired immune response.^204^

Furthermore, deleting α7nAChR in mice by increasing NF-κB resulted in elevated expression of profibrotic markers (α-SMA, collagen) and stimulated EndMT. *In vitro*, stimulating human cardiac microvascular endothelial cells (HCMECs) with an α7nAChR agonist reduced their transformation into myofibroblasts by inhibiting the IL-1β/NF-κB-dependent pathway.^205^ In rats undergoing coronary artery ligation, the administration of an α7nAChR agonist induced a reduction in the infiltration of monocytes and macrophages in the myocardium, resulting in an improved prognosis and reduced adverse cardiac remodelling.^206^ These studies demonstrate that cholinergic stimulation via α7nAChR regulates inflammatory cell activation and safeguards against excessive stimulation that leads to cardiac fibrosis.

Vagus nerve stimulation (VNS) in animal models gives promising results. Percutaneous VNS in Dahl salt-sensitive rats reduced blood pressure and significantly more effectively reduced cardiac remodelling than Olmesartan. Using methyllycaconitine, an α7nAchR antagonist, completely abolished the beneficial effects of VNS.^20^ VNS reduced the influx of inflammatory cells into the myocardium and the expression of IL-11, IL-18, OPN, and TNF-α.^207^ Additionally, in rats with heart failure due to thoracic aorta constriction, VNS not only reduced cardiac hypertrophy but also lessened inflammation intensity and microglial activation in the locus coeruleus and PVN.^208^ Recent RNA sequencing of the myocardium influenced by light stimulation of the optogenetically modified dorsal nucleus of the vagus nerve revealed differences in over 100 signalling pathways, along with the expression of transcription factors and miRNAs. Notable differences were observed concerning IL-2 transmission, the nuclear factor of activated T-cells (NFAT), and the eukaryotic initiation factor 2 (eIF2), all molecules associated with the production of pro-inflammatory cytokines during bacterial infections.^209^ These experiments demonstrate the strong connection between autonomic nervous system activation in immune regulation and myocardial remodelling. Moreover, it was shown that after MI in rats, significant autonomic hyperexcitability occurs, and methods using peripheral electrical nerve stimulation help maintain sympathetic-parasympathetic balance post-MI to improve heart function.^210^

There is limited evidence regarding the mechanisms of stimulation of other muscarinic receptors and the non-neuronal cholinergic system (NNCS), which is associated with the presence of choline transporter 1 (CHT1) and choline acetyltransferase (ChAT) alongside muscarinic receptors in cardiomyocytes. Previously, it was discovered that in mice following MI, overexpression of ChAT and hypoxia-inducible factor 1-alpha (HIF-1α) leads to reduced oxygen utilization by cardiomyocytes with increased glucose metabolism, which correlates with improved haemodynamic parameters and limited cardiac remodelling.^211^ Recently, it was also shown that rat cardiomyocytes subjected to hypoxic preconditioning displayed spontaneous increases in acetylcholine (Ach) secretion as a protective mechanism against cardiac injury and fibrosis.^212^ Interestingly, blocking CHT1 in healthy mice, despite a lack of haemodynamic changes at rest, resulted in excessively rapid heart rates and cardiomyocyte hypertrophy and fibrosis following exercise.^213^ This is related to the observation that physical exercise has a cardioprotective effect on the heart through cholinergic stimulation. Eight weeks of aerobic exercise in mice was associated with increased expression of muscarinic receptor type 2 (M2AChR) in cardiomyocytes and reduced oxidative stress and inflammation by inhibiting the NLRP3/caspase-1/IL-1β signalling pathway, in addition to fibrosis through downregulation of the PERK/eIF2α/ATF4 pathway.^214^ The application of an M2AChR inhibitor increased phosphorylation and activation of PERK/eIF2α/ATF4, abolishing exercise-induced cardioprotection in a myocardial ischaemia-reperfusion model in rats.^215^ In the fibrotic heart, increased expression of muscarinic receptor type 3 (M3AChR) was also noted, with stimulation inhibiting TGF-β1/Smad2/3 signalling and the non-canonical p38MAPK pathway, thereby protecting against their excessive activation.^216^

**Figure 3:**
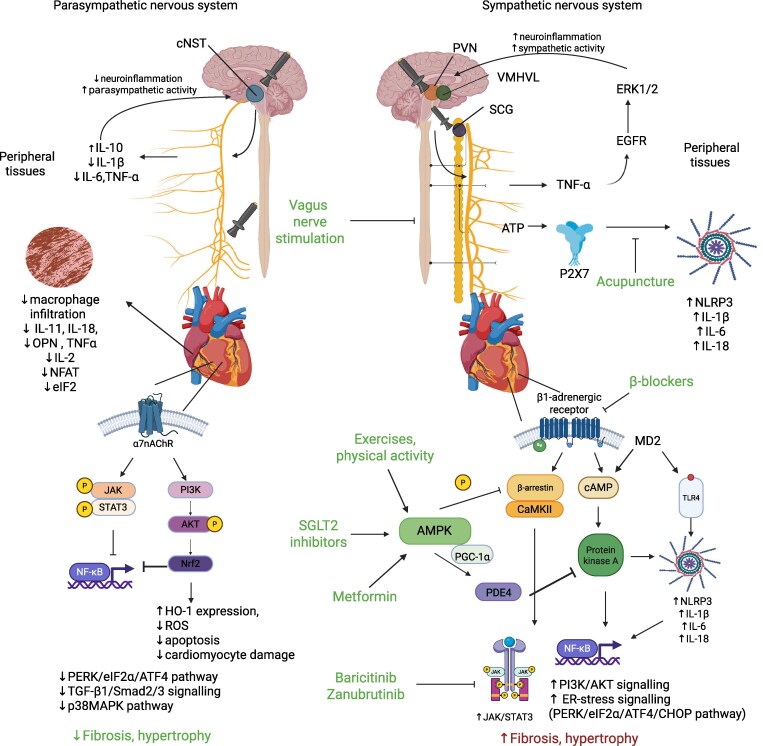
Neurostimulation of the sympathetic and parasympathetic nervous system and its effect on activating profibrotic and antifibrotic signalling pathways. Stimulation of sympathetic centres in the brain leads to increased peripheral inflammation. Additionally, patterned stimulation of the β-adrenergic receptor activates profibrotic mechanisms that depend on cAMP/PKA and β-arrestin/CAMKII, which are abolished by β-blockers, SGLT2 inhibitors, and metformin. Conversely, stimulation of parasympathetic centres and vagus nerve stimulation protects against excessive peripheral inflammatory responses, while the non-cholinergic nervous system in the heart reduces MyoFB activation. This figure has been created with Biorender.com. VMHVL, ventromedial hypothalamus; PVN, hypothalamic paraventricular nucleus; SCG, superior cervical ganglion; cNST, caudal nucleus of the solitary tract; ATP, adenosine triphosphate; P2X7, P2X purinoreceptor 7; NFAT, nuclear factor of activated T-cells; eIF2, eukaryotic initiation factor 2; OPN, osteopontin; NLRP3, nucleotide oligomerisation domain-like receptor family pyrin domain containing 3; NF-κB, nuclear factor kappa-light-chain-enhancer of activated B cells; SGLT2, sodium-glucose co-transporter 2; HO-1, haeme oxygenase-1; ROS, reactive oxygen species; AMPK, 5'AMP-activated protein kinase; PGC- 1 α, peroxisome proliferator- activated receptor gamma coactivator 1- alpha; PDE4, phosphodiesterase 4; CAMKII, Ca^2+/^calmodulin-dependent protein kinase II; TLR, toll-like receptors; p38 MAPK, p38 mitogen activating protein kinase; PI3K, phosphatidylinositol kinase; Nrf2, nuclear factor erythroid 2-related factor 2; TNF-α, tumour necrosis factor α; PERK, double stranded RNA activated protein kinase, like endoplasmic reticulum kinase; Erk1/2, extracellular signal-regulated kinase; EGFR, epidermal growth factor receptor; MD2, myeloid differentiation factor 2; α7nAChR, α7-nicotinic acetylcholine receptor.

*Figure 3* illustrates the neurostimulation of both the sympathetic and parasympathetic nervous systems and its effects on activating profibrotic and antifibrotic signalling pathways.

### 4.3 Neuroimmunoregulation of SGLT2 inhibitors

It is also worth briefly mentioning sodium- glucose co- transporter 2 (SGLT2) inhibitors, which serve as antidiabetic drugs that increase urinary glucose excretion and have recently become one of the fundamental groups of drugs improving the prognosis of HF or hypertrophic cardiomyopathy.^217^ Despite their widespread use, the mechanisms behind the pleiotropic effects of SGLT2 inhibitors on cardiomyocytes and CF are not fully understood. One recent meta-analysis comparing the effects of SGLT2 inhibitors in patients with T2DM and HFpEF observed a significant reduction in fibrosis, systolic blood pressure, and body weight, leading to improved left ventricular ejection fraction (LVEF) and better results in the 6-min walk test.^218^ Flozins also demonstrate high efficacy in non-diabetic patients with HFrEF. Six months of empagliflozin use in the EMPA-TROPISM study resulted in a significant reduction in ECM volume as shown by magnetic resonance imaging.^219^  *In vitro* studies on cardiomyocytes indicated that dapagliflozin reduced isoproterenol-dependent cardiomyocyte hypertrophy. In aortic endothelial cells, it inhibited the NLRP-3 inflammasome and limited the synthesis of pro-inflammatory cytokines, including TNF-α through the Akt/PI3K/MAPK signalling cascade.^220^ Additionally, flozins activate AMP-activated protein kinase (AMPK) and peroxisome proliferator-activated receptor gamma coactivator 1-alpha (PGC-1 α), reducing oxidative stress and reactive oxygen species (ROS) production.^221^ A Mendelian randomization study indicated that the chemokine CXCL10 has the strongest association among immune cells in mediating the effects of SGLT 2 inhibition in heart failure.^222^ Proteomic analysis within the EMPEROR programme revealed a strong correlation between empagliflozin (EMPA) use and a reduction in the expression of profibrotic and anabolic cellular messengers, which regulate interactions between proteins such as insulin-like growth factors 1 and 4 (IGF-1, IGF-4), protein-glutamine gamma-glutamyltransferase 2 (TGM 2), and adipocyte fatty acid-binding protein 4 (FABP 4) secreted by adipocytes and macrophages.^223^ Molecular analysis highlighted the impact of EMPA on Frizzled Class Receptor 4 (FZD 4), which regulates the profibrotic Wnt/β- catenin signalling pathway. Treatment with EMPA in transaortic constriction (TAC)-induced hypertrophy, along with *in vitro* studies in rat cardiomyocytes, attenuated the expression of the profibrotic Wnt/β-catenin pathway, thereby reducing cardiomyocyte hypertrophy and fibrosis.^224^

## 5. Epigenetic regulations—DNA and histone modifications in immune and fibrotic pathways

Recently, there has been increasing attention to the epigenetic regulation of both pre- and post-transcriptional gene expression related to DNA methylation, histone acetylation, and methylation, as well as the roles of non-coding RNAs (ncRNAs), including microRNAs (miRNAs), circular RNAs (circRNAs), and long non-coding RNAs (lncRNAs). Research indicates that during fibrosis and the progression of heart failure (HF), the expression of DNA methyltransferases (particularly DNMT1, DNMT3a, and DNMT3b) increases. This leads to hypermethylation of suppressor of cytokine signalling 3 (SOCS3), Ras-related domain family 1 (Rassf1) and Hedgehog signalling, thereby intensifying cardiac fibrosis profiling.^225,226^ Through hypermethylation of the peroxisome proliferator-activated receptor (PPAR_ϒ_1), the pro-M2 transcription factor, DNMT1 enhanced macrophage M1 polarization, increasing inflammation and fibrosis.^227,228^ Additionally, in an animal model the overexpression of DNMT3A, which leads to hypermethylation and the suppression of the lncRNA Neat1, stimulates the NLRP3 inflammasome, activating CF pyroptosis.^229^ Similar results were observed in the myocardium of ISO-fibrotic mice, where an increase in DNMT1, NLRP3, and caspase-1 expression occurred while silencing DMNT1 attenuated these processes.^230^ 5-Aza-2-deoxycytidine (DAC), an inhibitor of DNA methylation, caused proteome alteration in rats and reduced cardiomyocyte hypertrophy.^231^ Ten-eleven translocation (TET), particularly TET2 and TET3, enzymes responsible for DNA demethylation, has cardioprotective and antifibrotic properties. It has been shown that in the fibrotic heart, TET3 expression is reduced, resulting in DNA damage and activation of TGF-β signalling.^232^

Histone acetylation by histone acetyltransferases (HATs) relaxes chromatin, facilitating the interaction of transcription factors with DNA, while histone deacetylases (HDACs) result in a condensed form. The most recognized HAT in the pathogenesis of cardiac fibrosis is p300, which integrates collagen I and Smad2/Smad3, activating the TGF-β pathway and increasing fibrosis.^233^ The naturally occurring p300 inhibitor curcumin inhibits CF activation, and its analogues are presently undergoing testing in animal studies.^234^ Similar to HAT, most HDAC subgroups also exhibit profibrotic effects.^235^ The administration of LMK235, a selective HDAC4/5 inhibitor, to the MI-mice model inhibited TGF-β expression in CF and reduced cytokine secretion from macrophages by inhibiting lysine-specific demethylase 1 (LSD1)/NF-κB signalling cascade.^236^ It has been demonstrated *in vitro* in CF and *in vivo* in HFpEF mice that ischaemia increases HDAC6 expression.^237,238^ HDAC6-deficient (HDAC6^−/−^) mice after MI showed reduced expression of collagen I and III, TGF-β, phosphorylated Smad2/Smad3, correlating with a decreased infarct size and higher LVEF compared to the control group.^238^ In HFpEF mice, the administration of TYA-018, a selective HDAC6 inhibitor, reduced diastolic dysfunction and left ventricular hypertrophy while also decreasing the expression of profibrotic genes such as PDGFRα, TIMP1, and MMP2/MMP17, demonstrating results similar to those of EMPA treatment. TYA-018 increased the acetylation of troponin I, tropomyosin, and α-myosin heavy chain, directly affecting cardiomyocyte myofilaments. Moreover, TYA-018 applied *in vitro* to human induced pluripotent stem cell-derived cardiomyocytes (iPSC-CMs) increased their respiratory reserve and resistance to ischaemia by improving mitochondrial function.^237^ HDAC6 inhibition also reduced ischaemia/reperfusion injury in diabetic rats by reducing myocardial TNF-α production.^239^ Increased expression of HDAC3, HDAC4, and HDAC6 in the myocardium was also associated with elevated levels of pro-inflammatory cytokines (IL-18, IL-1β, TNF-α), stimulation of NF-κB, and infiltration of immune cells expressing CD3, CD8a and CD11c. The use of Ricolinostat (HDAC6 inhibitor) reduced the severity of inflammatory and fibrotic processes.^240^ The novel HDAC1 and HDAC6 inhibitor Se-SAHA, administered to ISO-induced HF mice and *in vitro* to neonatal rat ventricular myocytes, reduced oxidative stress, increased the expression of haeme oxygenase-1 (HO-1) and superoxide dismutase 2 (SOD2), and diminished beclin-1 expression and autophagosome accumulation, thereby eliminating the harmful effects of ISO.^241,242^

Histone acetylation of lysine residues influences the activity of the bromodomain and extra-terminal (BET) family, which includes BRD2, BRD3, and BRD4. This subsequently activates pro-inflammatory and profibrotic pathways during HF, the most notable of which is the action of BRD4 (bromodomain-containing protein 4). It has been shown that the use of JQ1, a BRD4 inhibitor, in mice with pressure-overload-induced HF after MI, improves prognosis by inhibiting NF-κB and TGF-β signalling.^243^ Single-cell epigenomic analyses revealed that the regulation of the transcription factor Meox1 in fibroblasts is associated with the activation of these cells by TGF-β.^244^ A recent publication by Alexanian et al. showed that BRD4 in CX3CR1+ macrophages infiltrating the myocardium enhances Meox1 signalling via IL-1β, thereby stimulating fibrosis. The deletion of IL1β in CX3CR1+ macrophages reduced Meox1 expression similarly to BRD4 deletion and alleviated HF.^245^ Apabetalone, a BET inhibitor, tested in clinical trials, shows various anti-inflammatory effects by reducing TNF-α, IL-1β or LPS-dependent activation of monocytes and their adhesion to the endothelium.^246^ In patients with type 2 diabetes and CVD treated with apabetalone, reduced cytokine secretion from monocytes and reduced TLR expression were observed.^247^ Potent anti-inflammatory and cardioprotective properties of BET inhibitors (BETi) were also confirmed in mice infected with SARS-CoV-2, where BETi protected cardiomyocytes against cytokine storm and reduced their apoptosis.^248^

Histone methylation most frequently affects arginine and lysine residues of histones H3 and H4 and is associated with the profibrotic effects of Ang II and endothelin expression.^249^ TGF-β stimulation in mice activated protein arginine methyltransferase 5 (PRMT5)/Smad3 complex, increasing α-SMA expression and worsening cardiac dysfunction. PRMT5 deletion in murine cardiomyocytes blocked TGF-β-dependent α-SMA expression, emphasizing the role of crosstalk histone methylation.^250^ In the mice model of MI, the administration of an inhibitor of the pro-inflammatory autotaxin (ATX)/lysophosphatidic acid (LPA) axis downregulated PRMT5, suggesting a role for inflammation in the activation of PRMT5 and TGF-β-dependent fibrosis.^251^ Histone lysine demethylases (KDM) also have strong profibrotic properties. KDM3A is responsible for the increased expression of TIMP1. KDM3A knockout mice or a pan-KDM inhibitor reduced cardiac hypertrophy induced by pressure in mice.^252^ Similar results were obtained by blocking KDM5B, which demethylates activating transcription factor 3 (Atf3), inhibiting its antifibrotic activity and increasing TGF-β signalling.^253,254^

*Table 3* summarizes the epigenetic regulation of DNA methylation, histone methylation, and acetylation regarding the expression and activation of pro-inflammatory and profibrotic signalling pathways.

**Table 3:** DNA methylation, histone methylation and acetylation in pro-inflammatory and profibrotic signalling pathways

Epigenetic process	Enzymes	Molecular mechanisms	Human/animal research
DNA methylation	DNA methyltransferases (**DNMT)**	↑PPAR_ϒ_ and M1 macrophages polarization^227^ ↓ lncRNA Neat1^229^ ↑ NLRP3 inflammasome^230^ ↓sFRP3, ↑Wnt/β-catenin pathway^255^ ↑SOCS3^225^ ↑Hedgehog signalling^226^	↓Cardiac hypertrophy in rats treated by DAC^231^ ↑DNMT1 expression promoted proliferation of mouse cardiac fibroblasts *in vitro*^227^
DNA demethylation	Ten-eleven translocation (**TET**) methylcytosine dioxygenases	↓RASAL promoter hypermethylation^256^ ↓IL-6^256^	↑CF in TET2 knockout mice after Ang II infusion^257^ ↓TET3 expression in fibrotic heart correlated with increased TGF-β signalling^232^
Histone acetylation	Histone acetyltransferases **(HATs)**/activity of the bromodomain and extra terminal **(BET)** family	↑Smad2/3, TGF-β signalling by p300^233^ ↑NF-κB^243^ ↑ Meox1 signalling via IL-1β in CX3CR1+ macrophages^244,245^	↓CF activation in mice^233^ ↓CF in mice with pressure-overload HF and after MI treated with BRD4 inhibitor^243^ ↓TNF-α, IL-1β secretion and TLR4 expression in patients treated with apabetalone^246^
Histone deacetylation	Histone deacetylases **(HDACs)**	↑Smad2/3, TGF-β signalling^238^ ↑ PDGFRα, TIMP1 expression^237^ **↑** IL-18, IL-1β, TNF-α expression and CD3, CD8a lymphocytes and CD11c monocytes inflitration in myocardium^240^ ↑NF-κB^236^ ↑ROS^241^ ↑p38 MAPK^258^ ↑TNF-α^239^	↓Collagen expression in HDAC-6 deficient mice after MI^238^ ↓Inflammation and CF proliferation after Ricolinostat treatment (HDAC-6 inhibitor)^240^ ↓Left ventricular hypertrophy in HFpEF mice after TYA-018 administration^237^ ↓ISO-induced HF in rats after Se-SAHA treatment (HDAC1/6 inhibitor)^241^
Histone methylation	Protein arginine methyltransferases **(PRMTs)**	↑Smad3 TGF-β signalling^250,251^	↓PRMT5 in CF limited left ventricular hypertrophy in pressure-overload mice^250^ ↓PRMT5 during reduction of inflammation after MI^251^
Histone demethylation	Histone lysine demethylases **(KDMs)**	↑Smad3 TGF-β signalling^253,254^ ↑TIMP1^252^ ↓Atf3^253^	↓KDM5B inhibited transition of CF to MyoFB^253^ ↓KDM3A and pan-KDM inhibitor alleviated pressure overload-induced fibrosis^252^

During heart failure, changes in the expression and activity of several enzymes involved in epigenetic modifications have been observed. Particular attention is given to the excessive activity of histone acetylases and deacetylases. Their activity stimulates the expression of pro-inflammatory cytokines and subsequently contributes to cardiac fibrosis. In animal models, blocking HDAC/HAT/BET shows promising results.

PPAR_ϒ_, peroxisome proliferator-activated receptor; sFRP3, secreted frizzled-related protein 3; SOCS3, suppressor of cytokine signalling 3; DAC, 5-Aza-2-deoxycytidine; CF, cardiac fibroblasts; PDGFRα, platelet-derived growth factor alpha; TIMP, tissue inhibitor of metalloproteinases; MI, myocardial infarction.; ISO, isoproterenol; Atf3, activating transcription factor 3.

## 6. Gut microbiota metabolites—among diet, inflammation, and CVD diseases

It has long been recognized that diet plays a crucial role in the development of hypertension and CVD. Currently, special attention is focused on changes in the gut microbiota (GM), secreted metabolites, maintenance of the gut barrier, and immunomodulation because the intestinal wall contains large amounts of gut-associated lymphoid tissue (GALT) and residual macrophages. GM stimulates dendritic cells, leading to the secretion of IgA by the intestinal mucosa and the production of antibacterial proteins—defensins, cathelicidins, and C-type lectins, which, by stimulating TLR receptors, help maintain immune balance. Furthermore, GM regulates the induction of anti-inflammatory myeloid-derived suppressor cells and pro-inflammatory Th17 lymphocytes.^259^ Obesity and hypertension lead to reduced GM diversity, an increased Firmicutes-to-Bacteroidetes ratio, a rise in populations of *Prevotella*, *Proteobacteria,* and *Klebsiella,* while decreasing commensal *Lactobacillus spp*. and increasing local inflammation.^260,261^ Mice on a high-salt diet exhibited increased inflammation in the gut wall, formation of IsoLG-protein adducts in CD11c+ dendritic cells and activation of lymphocytes due to increased expression of CD86. Additionally, faecal transplantation from hypertensive mice increased blood pressure in previously normotensive mice, emphasizing the role of GM in CVD development.^261^ Interestingly, recent studies have shown that changes in GM during HF contribute to an increased risk of cancer.^262^

Currently, for the treatment of hypertension and CVD, the DASH (Dietary Approaches to Stop Hypertension) diet and the Mediterranean diet are especially recommended due to their emphasis on consuming large amounts of fruits, vegetables, fibre, and whole grain products while limiting red meat, salt, sugar, and highly processed foods. In the SINFONI project, a randomized crossover-controlled study, CVD patients receiving a diet rich in bioactive compounds (slow-digestible starch, fibres, polyphenols) compared to the control group, showed a reduction in endotoxemia and intestinal inflammation measured by a decrease in fasting lipopolysaccharide and faecal calprotectin levels. Additionally, an increase in GM diversity was observed.^263^ A diet rich in fibre and GM activity promotes the production of short-chain fatty acids (SCFA), particularly butyrate, propionate, and acetate.^264^ A reduction in SCFA-producing bacteria has been demonstrated in patients with hypertension and hypertensive animal models.^265,266^ SCFAs bind to G protein-coupled receptors (GPCRs), particularly GPR41, GPR43, and GPR109A, and OLFR78 expressed on immune and epithelial cells, exerting anti-inflammatory effects.^267^ It has been shown that SCFA, through GPR43, increases the expression of Foxp3 in T lymphocytes, stimulating their differentiation towards Tregs, and SCFA administration to mice increases their concentration.^268^ Furthermore, SCFAs are HDAC inhibitors, increasing mTOR acetylation and inhibiting T-cell differentiation into pro-inflammatory Th1 and Th17 phenotypes.^269^ Propionate administered to Ang II-infused mice decreased splenic Th17 and memory T cells, which correlated with reduced hypertension and cardiac fibrosis. Interestingly, the depletion of Treg abrogated these results, emphasizing the critical role of T cells in the cardioprotective action of SCFAs.^270^ Sodium butyrate administered to hypertensive rats reduced BP by decreasing the expression of IL-1β and NLRP3 inflammasome.^271^ Furthermore, attenuation of NLRP3 inflammasome in atrial tissue via GPR43/NLRP3 signalling reduced atrial remodelling, collagen expression, and atrial fibrillation development in SCFA-treated mice.^272^ In mice treated with excess mineralocorticoids, high-fibre diet and acetate supplementation reduced RAAS activity in the kidney as well as TGF-β and MAPK signalling in the heart, resulting in decreased hypertension and cardiomyocyte hypertrophy.^266^ Some cardioprotective antioxidants, such as soy protein β-conglycinin have been shown to attribute their effects to SCFA. The administration of β-conglycinin increases SCFA-producing intestinal bacteria and reduces LV remodelling in pressure overload-induced heart failure, while depletion of bacteria through oral antibiotic administration abolishes these effects.^273^ Butyrate is used to treat inflammatory bowel disease, especially ulcerative colitis. The benefits of butyrate and other SCFAs should be considered alongside the potential to prevent the development of hypertension, cardiac fibrosis, and other conditions CVD.

In contrast to DASH and the Mediterranean diet, a diet based mainly on animal products provides more choline, phosphatidylcholine, L-carnitine, and betaine, which are subsequently converted into trimethylamine (TMA) by GM activity. TMA is metabolized in the liver and other tissues into trimethylamine N-oxide (TMAO), which has demonstrated profibrotic effects. Several previous studies have suggested a beneficial effect of low concentrations of TMAO in hypertensive rats, where TMAO exerts hypotensive, natriuretic, and mortality-reducing effects.^274,275^ Despite this, recent studies have highlighted a positive correlation between this GM metabolite and higher cardiovascular risk. A meta-analysis of nearly 7000 patients with heart failure revealed that elevated plasma TMAO concentrations over 5 years were linked to a greater risk of major adverse cardiac events and increased overall mortality.^276^ In the prospective cohort study, patients with HFrEF and HFpEF exhibited significantly higher TMAO levels compared to those without heart failure, demonstrating a better predictive value than NT-proBNP.^277^ Similar relationships have been observed in mice. A 3-week diet rich in choline and containing TMAO preceding surgical TAC led to increased cardiac fibrosis, left ventricular enlargement with elevated BNP levels, and a decrease in EF compared to the control diet.^278,279^ Moreover, in obese mice with increased plasma TMAO levels, the expression of IL-1β and TNF-α and decreased anti-inflammatory IL-10 were observed in the cardiac tissue.^280^  *In vitro* in cardiomyocytes, TMAO stimulated the expression of beta-myosin heavy chain and atrial natriuretic peptide, which were blocked by the administration of a Smad3 inhibitor, suggesting an effect of TMAO on TGF-β signalling.^281^ Moreover, in cardiac fibroblasts, TMAO stimulated the NLRP3 inflammasome.^282^ Interestingly, administering iodomethylcholine, which blocks the intestinal conversion of TMAO, to mice on a high-choline diet reduced cardiac remodelling, emphasizing the importance of GM activity in cardiac fibrosis.^283^ Recent publications have emphasized the significance of TMAO in aortic valve calcification and fibrosis, triggering ER stress, NF-κB signalling, and the *in vitro* expression of collagen I and TGF-β1 through the activation of the profibrotic IRE-1α/XBP-1 and PERK/ATF-4 pathways.^284–286^ TMAO also impairs aortic relaxation by blocking transient receptor potential vanilloid 4 (TRPV4) in the endothelium, inhibiting calcium transport and vasodilation, which increases the risk of hypertension.^287^ Elevated plasma TMAO levels in patients after ST-elevation MI have also been shown to correlate with a higher risk of atrial fibrillation and decreased LVEF.^288^ These studies highlight TMAO as an essential marker of CVD closely linked to gut microbiota.

Polysulfides and persulfides are vital dietary components with cardioprotective properties, primarily reliant on GM, especially in garlic, onions, and broccoli. These are reactive sulfane sulfur (RSS) compounds containing two sulfur atoms connected by a covalent bond, which provide potent antioxidant, electrophilic, and nucleophilic properties effective in neutralizing reactive oxygen species.^289^ It has been shown that after MI and in hypoxic conditions, RSS levels decrease, leading to increased oxidative stress and cellular damage.^290^ RSS is a source of hydrogen sulfide (H_2_S), a toxic gas with cardioprotective properties at low physiological concentrations produced independently by gut microbiota. In blood vessels, H_2_S acts synergistically with nitric oxide (NO), activating potassium channels, leading to vasodilation and a reduction in hypertension.^291^ A high-choline diet in spontaneously hypertensive rats, which increases the level of TMAO, resulted in a decrease in plasma H_2_S concentration and cystathionine-gamma-lyase (CSE) activity, the primary enzyme responsible for H_2_S synthesis in the heart. This leads to hypertrophy and increased collagen expression in cardiomyocytes, which is decreased by administering sodium hydrosulfide (NaHS), an H₂S donor.^292^ NaHS treatment reduced the expression of IL-1β, caspase-1, NLRP3 inflammasome, and stimulator of interferon genes STING in heart tissue, exerting anti-inflammatory effects and reversing the adverse impacts of TMAO.^293^ Moreover, NaHS increased sirtuin3 (SIRT3) sulfhydration *in vitro*, attenuated the pro-fibrotic effect of Ang II on the TGF-β1/Smad2/3 cascade and reduced atrial fibrosis *in vitro* and in rats with AF.

Furthermore, endothelial-cell-specific CSE knockout mice after TAC showed a significant increase in EndMT in cardiomyocytes, reduced NO synthesis, and worse exercise capacity compared to the control group, which showed CSE overexpression.^294^ These reports suggest that H_2_S donors may also potentiate and enhance the effects of SGLT2 inhibitors in HFpEF therapy.^295^ The effects of H_2_S and RSS in experimental studies are auspicious. Nevertheless, a significant issue arising from the labile structure of RSS is synthesizing a donor that can replicate the biological effects of RSS. A good example is naturally occurring compounds such as allicin or diallyl disulfide present in garlic, which also maintain the intestinal microbiota balance.^296^

**Figure 4:**
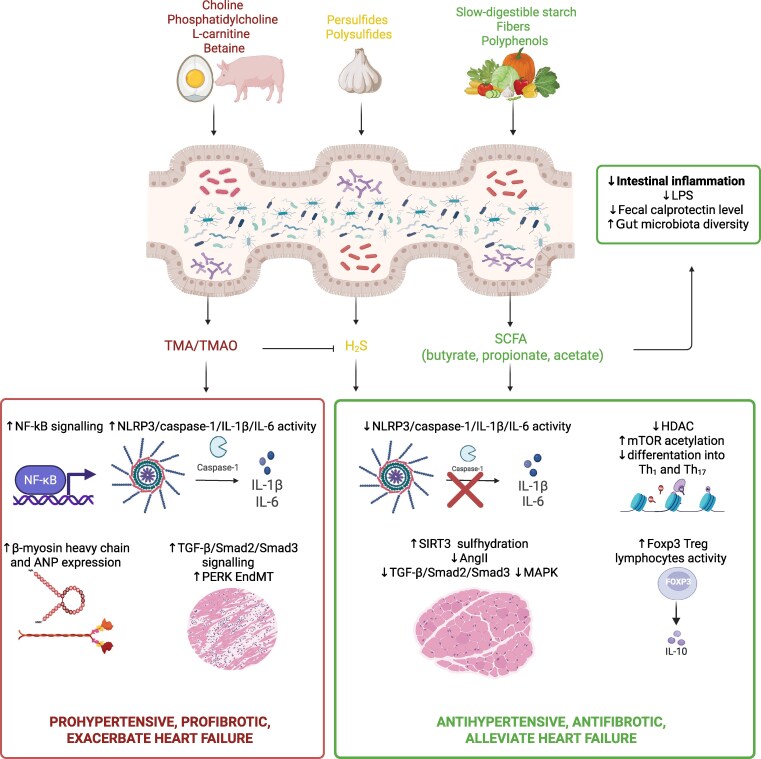
The influence of diet on gut microbiota diversity and its metabolites on the activation of pro-inflammatory and profibrotic processes during CVD development. The DASH and Mediterranean diets are recommended for treating hypertension. A diet rich in polysulfides and persulfides can also enhance gut microbiota diversity. Through SCFA and H_2_S metabolites, these diets reduce local inflammation, inhibit NLRP3 inflammasome and pro-inflammatory cytokine synthesis, and increase the activity of Treg lymphocytes. Additionally, they directly inhibit signalling cascades responsible for fibrosis. In contrast, a choline-rich diet lowers gut microbiota diversity and increases the risk of cardiovascular diseases through TMAO. This figure has been created with Biorender.com. SCFA, short-chain fatty acids; TMA, trimethylamine; TMAO, trimethylamine N-oxide; H_2_S, hydrogen sulfide; LPS, lipopolysaccharide; SIRT3, sirtuin 3; MAPK, mitogen activating protein kinase; HDAC, histone deacetylases; mTOR, mammalian target of rapamycin; EndMT, endothelial to mesenchymal transition; AngII, angiotensin II; ANP, atrial natriuretic peptide; NF-κB, nuclear factor kappa-light-chain-enhancer of activated B cells; NLRP3, nucleotide oligomerisation domain-like receptor family pyrin domain containing 3; TGF-β, transforming growth factor beta; PERK, double stranded RNA activated protein kinase—like endoplasmic reticulum kinase.

*Figure 4* summarizes the impact of diet, gut microbiota diversity, and their metabolites on the development of CVD.

## 7. Bone marrow and HF

Recently published papers emphasize the relationship between bone marrow activation and the development of HF.^297–299^ Evidence indicates that increased production of pro-inflammatory cytokines in the myocardium, including IL-6, IL-1β, and TNF-α, enhances myelopoiesis in the bone marrow and exacerbates the systemic inflammatory response.^300^ Moreover, pro-inflammatory haematopoiesis is further promoted by activation of the sympathetic nervous system and by endothelial dysfunction. In experimental models, blockade of adrenergic neurotransmission in mice reduced the mobilization of haematopoietic stem and progenitor cells. In contrast, administration of a β-AR agonist increased their release into the circulation.^301^ Notably, bone marrow transplantation from HF mice into healthy recipient mice resulted in spontaneous cardiac dysfunction and myocardial fibrosis.^302^

The role of age-related clonal haematopoiesis of indeterminate potential (CHIP) also warrants particular attention. Meta-analyses have demonstrated that CHIP is associated with a 25% increased risk of HF.^299^ In addition, patients with HF and coexisting CHIP exhibit a higher risk of hospitalization, HF-related mortality, and all-cause mortality.^303–305^ The strongest associations have been observed with mutations in genes involved in epigenetic regulation, including TET2, DNMT3A (discussed in Chapter 5), and JAK2 (Janus kinase 2).^299^ Preclinical studies targeting epigenetic regulators or selectively eliminating mutant haematopoietic clones offer promising perspectives for the future use of CHIP-directed strategies in the treatment of age-related cardiovascular diseases.^298^

## 8. Discussion and future perspectives

A multifactorial approach to treating HF is essential, as previous studies have revealed several severe adverse effects related to blocking individual pathways involved in the pathogenesis of HF. As mentioned above, attempts to block individual cytokines such as IL-1β in the CANTOS study, IL-6, or T lymphocytes, while reducing hypertension and cardiovascular risk, were associated with several adverse effects that significantly increased susceptibility to severe infections, outweighing the benefits of the therapy applied. Similarly, blockade of TGF-β transmission led to impaired wound healing and the formation of ruptures in the myocardium. It is impossible to block sympathetic transmission entirely because it provides the appropriate strength and electrical transmission in cardiomyocytes for proper contraction. For this reason, it is crucial to thoroughly understand the molecular interactions among individual ECM and MyoFB proteins, immune cells, epigenetic mechanisms and how they change in response to neural stimulation. The possibility of blocking more specific and single molecules that would not block the entire signalling pathway could reduce the risk of severe adverse effects.

Additionally, emphasis is placed on designing nanoparticles that can transport drugs and therapeutic compounds to the targeted site of action, thereby minimizing side effects in other organs. An example is CAR-T therapy, which is being tested in cardiac fibrosis. This therapy involves appropriately modifying T lymphocytes to act strictly on active MyoFB and locally limit inflammation and profibrotic signalling cascades. In neuroimmunomodulation, research is focused on designing bioelectronic devices that can safely and effectively modulate the peripheral immune response.

Another major disadvantage is the limited number of studies confirming the molecular mechanisms of HF development in humans. Most experimental studies originate from research conducted on animals. For ethical reasons, it is challenging to perform similar experiments *in vivo* with the participation of humans. Nevertheless, studies showing differences in the expression of individual ECM proteins, inflammatory cells and epigenetic changes from the hearts of patients with HF undergoing transplantation would be very valuable. It would be crucial to compare these changes depending on the cause of HF (e.g. ischaemic HF, hypertensive HF, diabetic cardiomyopathy, hypertrophic cardiomyopathy). Moreover, these data enabled us to determine whether individual ECM proteins and MyoFB markers could serve as biomarkers to help assess diagnosis, prognosis, and treatment efficacy. Furthermore, the possibilities of their application in modern diagnostic imaging methods, including evaluating cardiac hypertrophy in magnetic resonance imaging, should be investigated.

Finally, the data underline that diet, notably the DASH and Mediterranean diets, and physical exercise directly affect the molecular mechanisms involved in developing hypertension and cardiac fibrosis. They are also easy to implement and lack significant side effects.
